# Combined inhibition of MTAP and MAT2a mimics synthetic lethality in tumor models *via* PRMT5 inhibition

**DOI:** 10.1016/j.jbc.2023.105492

**Published:** 2023-11-23

**Authors:** Gabriel T. Bedard, Nord Gilaj, Karina Peregrina, Isabella Brew, Elena Tosti, Karl Shaffer, Peter C. Tyler, Winfried Edelmann, Leonard H. Augenlicht, Vern L. Schramm

**Affiliations:** 1 Department of Biochemistry, Albert Einstein College of Medicine, Bronx, New York, USA; 2 Department of Chemistry, Lehman College, Bronx, New York, USA; 3 Department of Oncology, Albert Einstein College of Medicine, Bronx, New York, USA; 4 Department of Medicine, Albert Einstein College of Medicine, Bronx, New York, USA; 5 Department of Cell Biology, Albert Einstein College of Medicine, Bronx, New York, USA; 6 Department of Genetics, Albert Einstein College of Medicine, Bronx, New York, USA; 7 Ferrier Research Institute, Victoria University of Wellington, Lower Hutt, New Zealand

**Keywords:** MTAP, MAT2a, PRMT5, colorectal cancer, cancer metabolism, arginine methylation, *S*-adenosyl-L-methionine, 5'-methylthioadenosine

## Abstract

Homozygous 5′-methylthioadenosine phosphorylase (*MTAP*) deletions occur in approximately 15% of human cancers. Co-deletion of *MTAP* and methionine adenosyltransferase 2 alpha (*MAT2a*) induces a synthetic lethal phenotype involving protein arginine methyltransferase 5 (PRMT5) inhibition. MAT2a inhibitors are now in clinical trials for genotypic *MTAP*^*−/−*^ cancers, however the *MTAP*^*−/−*^ genotype represents fewer than 2% of human colorectal cancers (CRCs), limiting the utility of MAT2a inhibitors in these and other *MTAP*^*+/+*^ cancers. Methylthio-DADMe-immucillin-A (MTDIA) is a picomolar transition state analog inhibitor of MTAP that renders cells enzymatically MTAP-deficient to induce the *MTAP*^*−/−*^ phenotype. Here, we demonstrate that MTDIA and MAT2a inhibitor AG-270 combination therapy mimics synthetic lethality in *MTAP*^+/+^ CRC cell lines with similar effects in mouse xenografts and without adverse histology on normal tissues. Combination treatment is synergistic with a 10^4^-fold increase in drug potency for inhibition of CRC cell growth in culture. Combined MTDIA and AG-270 decreases *S*-adenosyl-L-methionine and increases 5′-methylthioadenosine in cells. The increased intracellular methylthioadenosine:*S*-adenosyl-L-methionine ratio inhibits PRMT5 activity, leading to cellular arrest and apoptotic cell death by causing MDM4 alternative splicing and p53 activation. Combination MTDIA and AG-270 treatment differs from direct inhibition of PRMT5 by GSK3326595 by avoiding toxicity caused by cell death in the normal gut epithelium induced by the PRMT5 inhibitor. The combination of MTAP and MAT2a inhibitors expands this synthetic lethal approach to include *MTAP*^*+/+*^ cancers, especially the remaining 98% of CRCs without the *MTAP*^*−/−*^ genotype.

Loss of the metabolic enzyme 5′-methylthioadenosine phosphorylase (MTAP, EC:2.4.2.28) is the most frequent metabolic gene deletion in human cancers due to its close (100 kb) sequence proximity to the *CDKN2A/B* locus (1). Genetic knockdown screening libraries revealed that in the 15% of cancers with MTAP loss, enhanced dependence is conferred on methylthioadenosine transferase 2 alpha (MAT2a, EC:2.5.1.6) and protein arginine methyltransferase 5 (PRMT5, EC:2.1.1.320) (2, 3, 4). These synthetic lethal interactions between MTAP loss and MAT2a or PRMT5 knock-down have driven the development of MAT2a and PRMT5 inhibitors that are now in clinical testing for treatment of malignancies with the *MTAP*^*−/−*^ genotype (5, 6).

The MTAP, MAT2a, and PRMT5 gene products are functionally linked through their involvement in the methionine/*S*-adenosyl-L-methionine (SAM) cycle (Fig. 1*A*). MAT2a catalyzes the synthesis of SAM, an essential metabolite in polyamine biosynthesis and methylation reactions. Spermine production from SAM produces two equivalents of 5′-methylthioadenosine (MTA), which requires MTAP for recycling to methionine. SAM is also used as a cofactor for methyltransferases, including PRMT5, that mediate regulatory post-translational and epigenetic methylations on proteins and DNA. SAM-consuming methyltransferases contain adenosyl-recognition motifs that can be inhibited by adenosyl-metabolites including SAH or MTA. Regulation of inhibitory metabolites is critical for maintaining cellular homeostasis; therefore methyl-metabolite pool disruption can have tumor suppressing activity if alterations affect the activity of onco-driving methyltransferases.

**Figure 1:**
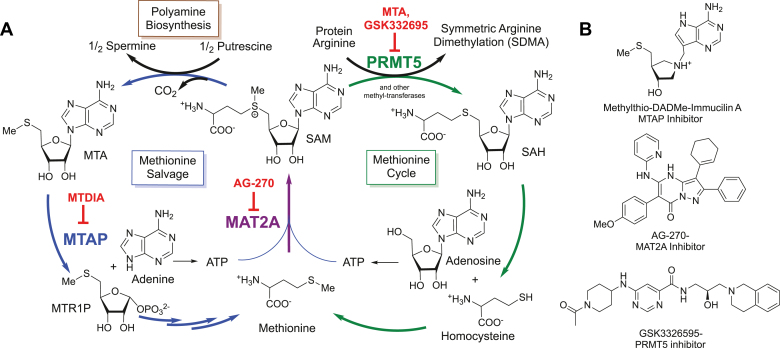
**Metabolic pathway of SAM recycling and pathway inhibitors.***A*, methionine salvage and cycle pathways. *B*, chemical structures for inhibitors MTDIA, AG-270, and GSK3326595. MTDIA, methylthio-DADMe-immucillin-A; MTR1P, methylthioribose-1-phosphate; SAH, *S*-adenosyl-L-homocysteine.

PRMT5 is a transformation-driving type II arginine methyltransferase responsible for the majority of protein symmetric dimethylarginine (SDMA, also Rme2s) post-translational modifications (7, 8, 9, 10, 11). PRMT5 catalyzes the vast majority of SDMA posttranslational modifications on histones (12), splicing factors (13), transcription factors and enhancer-binding proteins (14, 15), metabolic enzymes, and cell signaling proteins (16, 17, 18) implicating its role in cancer progression and survival. PRMT5 is overexpressed and negatively correlated with survival in patients with colorectal cancer and other cancers (19). PRMT5 is unique among methyltransferases in that its affinity for SAM is 40-times weaker than its affinity for MTA (SAM *K*_M_ = 10.3 μM; MTA *K*_i_ = 0.26 μM) making PRMT5 uniquely sensitive to metabolite pool sizes of MTA and SAM (2). Consequently, in *MTAP*^*−/−*^ tumors the synthetic lethal inhibition of MAT2a ultimately converges on PRMT5 inhibition that reduces cancer growth and induces cell death.

Challenges for the use of MAT2a inhibitors include the variable frequency of the *MTAP*^*−/−*^ genotype, which ranges from 55% prevalence in glioblastoma to 2% in colorectal carcinoma, limiting the broader application of these therapeutics (20). MTAP deletion is linked to the mechanism of action requiring the accumulation of MTA within the tumor. However, the MTA cellular content of *MTAP*^*−/−*^ tumors is reduced by efflux of MTA into neighboring stromal or infiltrating immune MTAP-competent cells limiting the intracellular MTA accumulation and decreasing the therapeutic window of MAT2a inhibitors (21). Therefore, the local cancer *MTAP*^*−/−*^ genotype may be suboptimal for clinical efficacy of MAT2a inhibitors.

We have developed picomolar transition state (TS) analogs of MTAP, including methylthio-DADMe-immucillin-A (MTDIA). MTDIA binds tightly to MTAP, causing MTA accumulation in blood and tissues of treated mice (22, 23) (Fig. 1*B*). MTDIA induces apoptosis in xenografts of head and neck squamous carcinomas, reduces growth and metastases in mice bearing subcutaneous lung carcinoma xenografts, and extends the lifespan of a familial adenomatous polyposis mouse model by significantly inhibiting tumor growth (24, 25, 26). MTDIA binds 58,000 times tighter than MTA to the MTAP active site to drive the accumulation of MTA. MTDIA shows oral availability, low toxicity, and high specificity for MTAP inhibition in the mouse (24, 26). Therefore, MTDIA is effective in mimicking the metabolic phenotype of *MTAP*^*−/−*^ cancers.

This work investigates the potential of MTDIA to induce the *MTAP*^*−/−*^ synthetic lethal phenotype in *MTAP*^*+/+*^ cancers when combined with MAT2a inhibitors. This approach differs from the tumor genetic deficiency of synthetic lethality that converges on PRMT5 inhibition. Here, the systemic metabolic increase of the MTA:SAM ratio suppresses the catalytic activity of PRMT5 activity, which alters tumor cells more than normal cells to mimic the synthetic lethal phenotype (9). This extension of the synthetic lethal approach to MTAP and MAT2a inhibition provides a new potential therapy for colorectal carcinomas (CRCs) as only 2% of human CRCs are *MTAP*^*−/−*^ (20). We hypothesized that MTDIA treatment would sensitize MTAP-competent CRCs to treatment with the MAT2a inhibitor AG-270 through the induction of a *MTAP*^*−/−*^ metabolic phenotype. The data herein demonstrate that MTDIA and AG-270 have synergistic effects in reducing cell growth *in vitro* through the increase in the MTA:SAM metabolite ratio that inhibits PRMT5 activity and resulting in downstream effects on RNA splicing activity, p53 activation, cell growth arrest, and tumor growth inhibition in murine xenograft models. Importantly, we demonstrate that systemic inhibition of MTDIA and AG-270 in mice does not induce cell death in normal gut mucosa in contrast to treatment with the PRMT5 inhibitor GSK3326595. The drug synergy afforded by combination MTDIA and AG-270 treatment suggests that MTDIA has the potential to expand the use of MAT2a inhibitors to CRCs and other cancers that are *MTAP*^*+/+*^.

## Results

### MTDIA and AG-270 inhibit cancer cell growth

**Figure 2:**
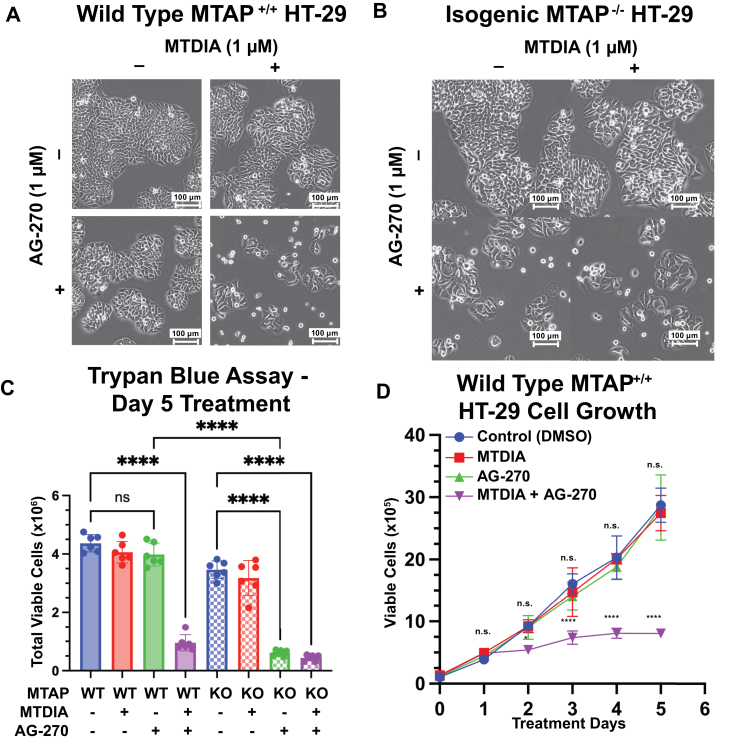
**Colon cancer cell (HT-29) growth response to MTAP and/or MAT2a inhibition.***A* and *B*, WT and isogenic MTAP KO HT-29 cells treated for 5 days with MTDIA and AG270 and visualized under phase-contrast bright field microscopy. *C*, WT and KO HT-29 cell counts after 5 days treatment with MTDIA and AG-270. Total viable cells determined by hemacytometer and trypan blue exclusion. Significance determined with one-way ANOVA. *D*, growth curve of WT HT-29 cells with 100,000 cells plated at day 0. All drugs used at 1 μM. Total viable cells determined by hemacytometer and trypan blue exclusion. MTAP, 5′-methylthioadenosine phosphorylase; MTDIA, methylthio-DADMe-immucillin-A.

Treatment of WT *MTAP*^*+/+*^ HT-29 cells or isogenic HT-29 *MTAP*^*−/−*^ cells generated by CRISPR KO (henceforth MTAP KO) with 1 μM MTDIA did not alter cell morphology or growth over a 5-day period evaluated by phase-contrast microscopy (27) (Fig. 2, *A* and *B*). AG-270 treatment alone (1 μM) did not alter growth or morphology of MTAP WT cells; however this induced a 5.7 ± 1.0-fold reduction in viability in MTAP KO cells under the same conditions and was accompanied by morphological changes including cell detachment, friable edges and multinucleate and sparse cell clusters throughout the dish. Combination treatment of 1 μM MTDIA and 1 μM AG-270 in MTAP WT cells led to a comparable 4.6 ± 1.4-fold reduction in viability and similar morphological changes after 5 days, suggesting that combination MTDIA and AG-270 mimicked the synthetic lethal phenotype observed in MTAP KO cells treated with AG-270 alone (Fig. 2*C*). As early as day two, treatment with MTDIA and AG-270 reduced viable cells by 1.7 ± 0.1-fold (*p* = 0.0381) and reduced cell growth progressively through day 5 (Fig. 2*D*). Treatment with GSK3326595, a peptide-competitive inhibitor of PRMT5, induced similar morphological and growth-inhibition phenotypes in both WT and KO cells (Fig. S2, *A*–*C*). When used in combination with MTDIA and AG-270, GSK3326595 did not enhance MTDIA + AG-270 growth inhibition effects (Fig. S2*D*), supporting PRMT5 as the convergent target for the observed phenotype.

Previous reports of MTDIA effects in cultured cells indicated growth inhibition only when MTA supplementation facilitated its intracellular accumulation (25). Here, 20 μM MTA alone in HT-29 cell media did not result in growth inhibition due to its rapid metabolism by MTAP. However, 20 μM MTA in combination with MTDIA caused moderate growth inhibition (2.1 ± 0.4-fold reduction at day five, *p* < 0.0001), less than that observed with the combination of MTDIA and AG-270 treatment. Growth inhibition with combined MTDIA and AG-270 treatment was not enhanced by 20 μM MTA (Fig. S2*E*).

### MTDIA and AG-270 are synergistic

**Figure 3:**
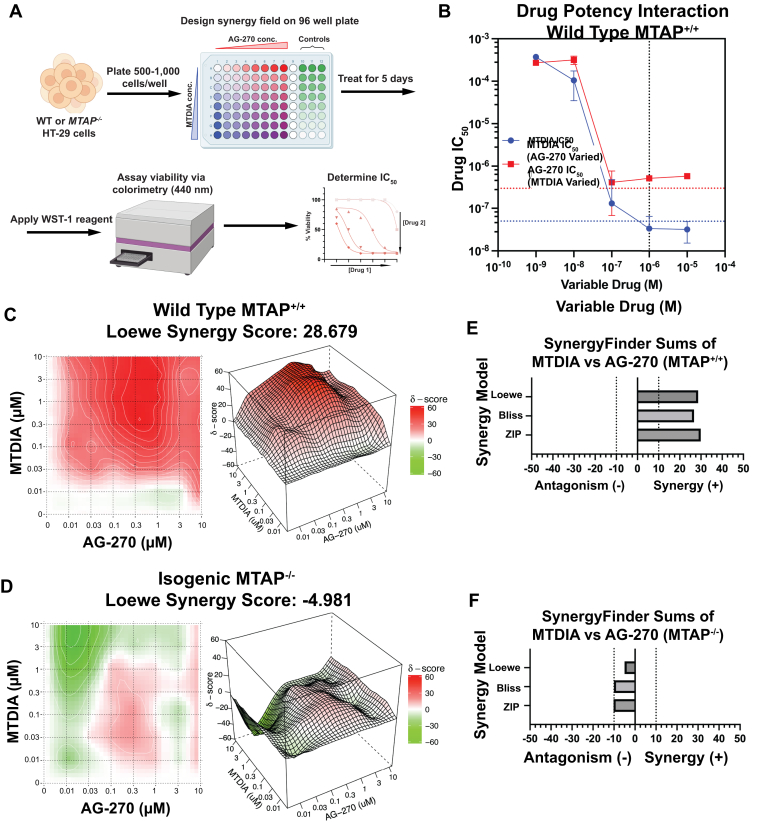
**Synergistic inhibition of HT-29 cell growth by MTDIA and AG-270.***A*, workflow for determining IC_50_ with drug combinations. *B*, IC_50_ analysis of MTDIA and AG-270 at varying concentrations of secondary drug in WT HT-29 cells. *Blue dotted horizontal line* represents IC_50_ values in the presence of 20 μM MTA, *red dotted horizontal line* represents IC_50_ value in MTAP^−/−^ cells, and *black vertical dotted line* indicates drug concentration used for further cell assays. IC_50_ determined by WST-1 assay after 5 days treatment. *C* and *D*, SynergyFinder3.0 analysis for drug synergy with MTDIA and AG-270 at 1 μM in WT (*E*) and isogenic KO (*F*) HT-29 cells using LOEWE, BLISS, and ZIP models. Positive scores (*red*) are synergistic and negative scores (*green*) are antagonistic. MTAP, 5′-methylthioadenosine phosphorylase; MTDIA, methylthio-DADMe-immucillin-A.

Pharmacologic synergism between MTDIA and AG-270 was investigated by a growth inhibition assay in a 96-well plate. Concentrations of MTDIA and AG-270 were varied in a comprehensive matrix from 1 nM to 10 μM to determine the concentrations of each drug that achieves 50% growth inhibition (IC_50_) alone or in the presence of the second drug (Fig. 3*A*). Individually, neither MTDIA nor AG-270 caused detectable growth inhibition of HT-29 WT MTAP cells (Fig. S3*A*), consistent with previously reported IC_50_ values >300 μM (28). With MTDIA at 1 μM, the AG-270 IC_50_ value decreased to 228 ± 15 nM, a synergistic shift in drug potency of approximately 1000-fold. Similarly, with AG-270 at 1 μM, the IC_50_ for MTDIA decreased to 56 ± 3 nM, a synergistic shift in drug potency of approximately 10,000-fold (Fig. 3*B*). SynergyFinder3.0 analyzed synergism using three independent models of drug synergy (Loewe, BLISS, and ZIP). Each model identified synergism at drug concentrations from 0.01 to 10 μM by varying drug combinations (Fig. 3, *C* and *E*). In contrast, no MTDIA and AG-270 synergistic interaction occurs in the MTAP KO cell line, establishing MTAP as an essential target for MTDIA (Figs. 3, *D* and *F* and S3, *A* and *B*). Synergy analysis of PRMT5 inhibitor GSK3326595 with MTDIA or in combination with MTDIA and AG-270 revealed no significant synergy landscape in HT-29 MTAP WT cells (Fig. S3, *C*–*G*). Thus, MTDIA recapitulates the *MTAP*^*−/−*^ synthetic lethal phenotype and synergizes with MAT2a inhibitor AG-270 for growth inhibition in *MTAP*^*+/+*^ cells.

### MTDIA and AG-270 in methionine metabolism

**Figure 4:**
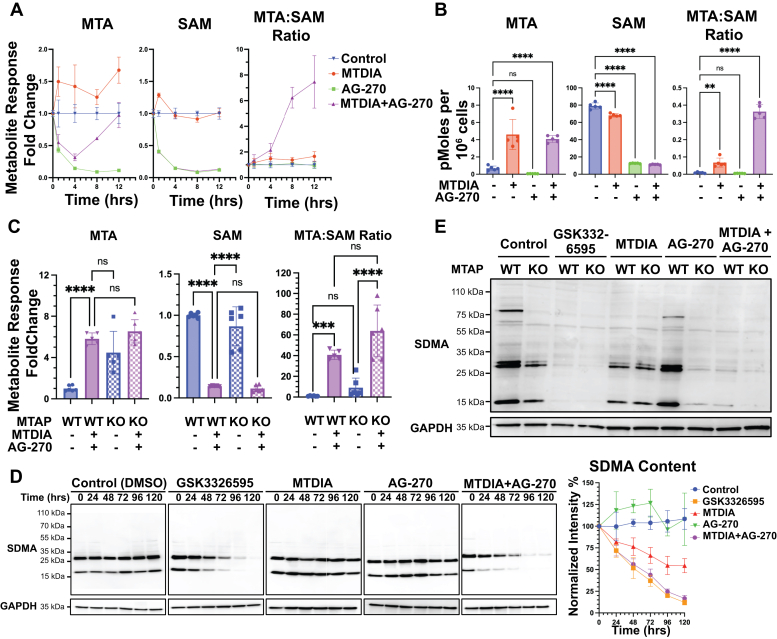
**MTDIA- and AG-270-induced changes in SAM metabolites and protein methylation.***A*, time-course treatment of MTA and SAM metabolic pool size alterations in WT HT-29 cells. Metabolite concentrations measured by LC-MS and normalized to relative signal of untreated control. *B*, quantified MTA and SAM metabolite pool sizes measured after 24 h treatment in WT HT-29 cells. *C*, comparison of WT and isogenic KO HT-29 metabolite pool sizes after 24 h of treatment. Significances determined by one-way ANOVA. *D*, time-course of loss of SDMA content in WT HT-29 cells by Western blotting. Twenty micrograms of protein loaded in each lane, and relative SDMA intensity normalized to GAPDH. Quantification performed on four biological replicates. *E*, endpoint analysis of WT and KO HT-29 cells after 5 days treatment in culture. MTA, 5′-methylthioadenosine; SDMA, symmetric dimethylarginine; SAM, *S*-adenosyl-L-methionine.

Inhibitor synergy between MTDIA and AG-270 suggests perturbation of intracellular metabolite pools. The effects of MTDIA, AG-270, and their combination on the metabolite pools of MTA, SAM, and related metabolites were determined. Metabolic changes occurred within 30 min of MTDIA and AG-270 treatment and persisted for up to 24 h (Fig. 4, *A* and *B*). Steady-state SAM levels in untreated HT-29 cells were 79 pmol per 10^6^ cells (approximately 20 μM) and MTA levels were 0.69 pmol per 10^6^ cells (approximately 17 nM, Figs. 4*B* and S4, *A*). MTDIA treatment did not affect the SAM pool, however AG-270 treatment decreased SAM by 6.3-fold to approximately 3 μM. MTDIA treatment caused accumulation of MTA inside the cell with a 5.5-fold increase to approximately 1 μM after 24 h of treatment. AG-270 treatment alone decreased cellular MTA pool by 8.9-fold to 2 nM in parallel with SAM depletion kinetics. The combination of MTDIA with AG-270 restored MTA to the level of MTDIA monotherapy in 24 h (Fig. 4, *A* and *B*). Cellular MTA content following MTDIA treatment in HT-29 cells is the same as in KO MTAP cells, consistent with metabolic equivalence for MTDIA treatment and genetic MTAP KO (Fig. 4*C*). The SAM pools remain the same in WT and MTAP KO HT-29 cells. In summary, inhibition or knockout of MTAP together with AG-270 in HT-29 cells caused an increase of 40 to 60-fold in the MTA:SAM ratio after 24 h (Fig. 4*C*).

Inhibition of either MTAP by MTDIA or MAT2a by AG-270 caused only modest changes in other methionine cycle–related metabolites, including SAH, homocysteine, methionine, putrescine, spermidine, and spermine (Fig. S4, *B* and *C*).

### MTA:SAM ratio modulates the activity of PRMT5

The reported *K*_i_ and *K*_M_ values for MTA and SAM (0.26 and 10 μM, respectively), together with their measured cellular concentrations (17 nM and 20 μM, respectively), can be used to estimate PRMT5 inhibition by MTA. Under normal physiological conditions, PRMT5 is 26% inhibited by MTA. After drug treatment, measurements of MTA indicate concentrations 5-fold greater than its reported *K*_i_ and SAM concentrations 3-fold less than its *K*_M_. Thus, PRMT5 is inhibited by 73% binding to MTA with MTDIA alone and over 93% inhibited with combination MTDIA and AG-270 treatment. PRMT5 catalyzes the majority of SDMA (11) and cellular SDMA content was estimated by Western blotting. Inhibition of PRMT5 by GSK3326595 in MTAP WT HT-29 cells led to >90% (*p* = 0.015) reduction of SDMA content in cells (Fig. 4*D*). MTDIA treatment alone caused 50% (*p* = 0.085) depletion of SDMA, consistent with increased PRMT5 inhibition under elevated MTA conditions. AG-270 treatment alone for 5 days did not affect cellular SDMA, however, the combination of MTDIA and AG-270 treatment led to >90% (*p* = 0.015) SDMA reduction at 5 days, similar to the effects of the PRMT5-specific inhibitor GSK3326595 (Fig. 4*D*).

MTDIA inhibition of MTAP was compared to MTAP KO for effects on PRMT5 inhibition as measured by SDMA content. MTDIA treatment and MTAP KO caused similar reductions in SDMA content in HT-29 cells after 5-day treatment with AG-270. AG-270 treatment of MTAP KO cells caused reduction in SDMA but the same treatment did not reduce SDMA in *MTAP*^*+/+*^ cells. Thus, the combination of MTDIA and AG-270 in MTAP WT cells is equivalent to AG-270 in MTAP KO cells with respect to the loss of SDMA (Fig. 4*E*).

### Cell line and PRMT5 specificity

**Figure 5:**
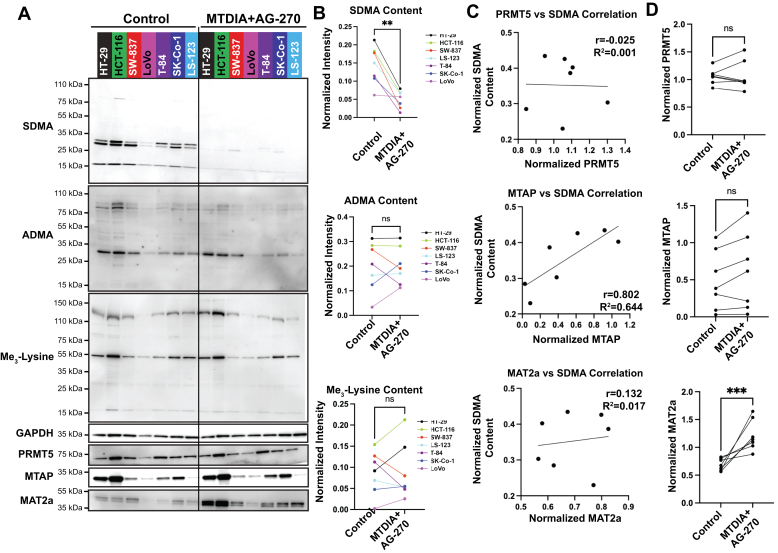
**Enzyme expression and protein methylation in CRC cell lines treated with MTDIA and AG-270.***A*, comparison of seven CRC cell lines after 5 days treatment in culture. Western blotting was performed on two biological replicates. *B*, quantification and comparison of symmetric dimethyl-arginine (SDMA, *p* = 0.0021), asymmetric dimethyl-arginine (ADMA, *p* = 0.9487), and trimethyl-lysine (*p* = 0.8954) content normalized to GAPDH. Quantification performed in ImageJ. Data shown here are the quantification of representative blots shown in *panel A*. Significance determined with Student’s *t* test. *C*, correlation analysis of untreated bands in *panel A*. Pearson’s coefficient and coefficient of determination calculated in Graphpad Prism. *D*, comparison of protein changes in *panel A* after 5 days treatment in culture. CRC, colorectal carcinoma.

CRC cell lines representing genotypes common in colon adenomas and carcinomas, including mutations in KRAS, APC, FAM123B, FBXW7, MSH2, PIK3CA, SMAD4, and TP53 were tested for their response to MTDIA and AG-270 combination treatment. Cell lines HT-29, HCT-116, LoVo, LS-123, SK-Co-1, SW-837, and T-84 were treated for 5 days. The cell lines demonstrated an average of 70 ± 20% reduction in global SDMA content (*p* = 0.0021 Fig. 5, *A* and *B*), demonstrating the combination of MTDIA and AG-270 treatment to be broadly applicable to CRC genotypes. The specificity of MTDIA and AG-270 treatment for PRMT5 was probed by Western blotting for asymmetric dimethylarginine (ADMA), catalyzed predominantly by PRMTs 1 to 4, and trimethyl lysine, catalyzed by several lysine methyltransferases. Combination MTDIA and AG-270 treatment had no global or band-specific effects on ADMA (*p* = 0.9487) or trimethyl lysine (*p* = 0.8954) content, demonstrating the high specificity for PRMT5 activity (Fig. 5, *A* and *B*).

Correlation analysis between cellular SDMA and PRMT5, MTAP, and MAT2a protein content showed no correlation between PRMT5 protein and SDMA (r = −0.025, R^2^ = 0.001), however MTAP positively correlated with SDMA content (r = 8.02, R^2^ = 0.644, Fig. 5*C*). No change in PRMT5 or MTAP protein content was observed after 5 days treatment with MTDIA and AG-270, but there was an increase in MAT2a protein in all seven cell lines (Fig. 5*D*).

### Growth arrest, apoptosis, and alternative RNA splicing

**Figure 6:**
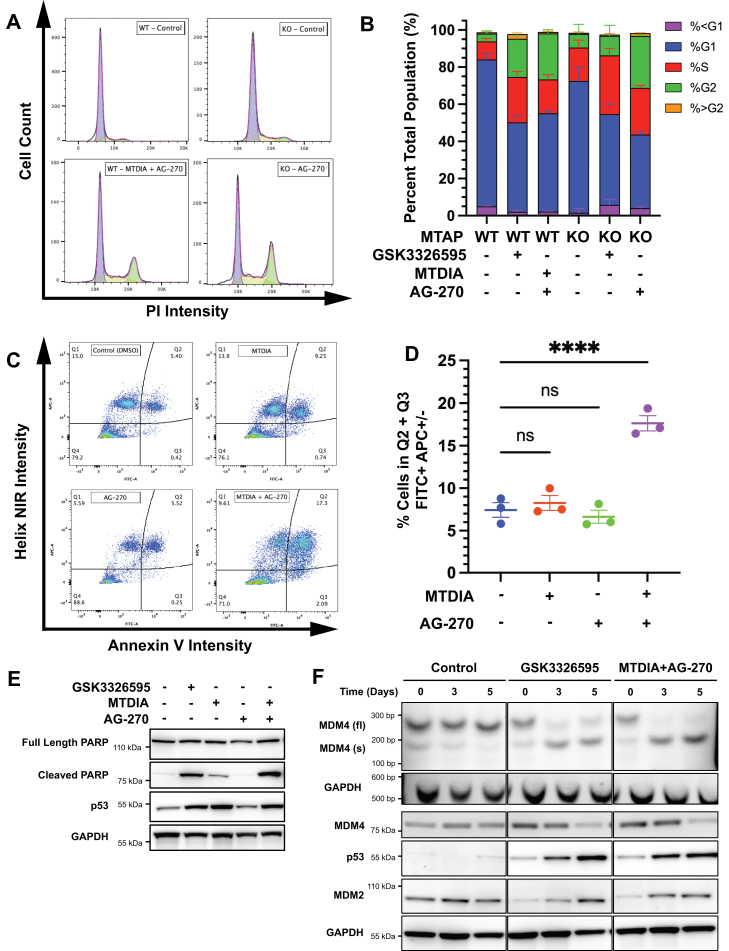
**Mechanism of cell growth inhibition induced by MTDIA and AG-270.***A* and *B*, representative cytometric traces analyzing cell cycle. Cells stained with propidium iodide (PI) and analyzed by Cellometer Spectrum fluorescent imager. Analysis performed in FlowJo (n = 4) and plotted in GraphPad prism. *C* and *D*, Representative flow cytometric gating analysis of WT HT-29 cells after 5 days treatment. Quantification of early (Q3) and late (Q4) apoptosis performed in FlowJo (n = 3) and plotted in GraphPad Prism. *E*, Western blotting of molecular markers of apoptosis in WT HT-29 cells after 5 days treatment. *F*, RT-PCR amplification of MDM4 full length (fl) and spliced (s) variants flanking exon 7 (*top*) and Western blotting of full-length MDM4, p53, and MDM2 (*bottom*) proteins in WT HT-29 cells.

PRMT5 loss, through either genetic inhibition or pharmacologic inhibition, causes alterations in RNA splicing and gene expression, resulting in growth inhibition and cell death (29, 30, 31, 32). Therefore, the effects of MTDIA and AG-270 were evaluated on cell cycle arrest, apoptosis, and specific splicing events associated with PRMT5 inhibition. Combination MTDIA and AG-270 treatment increased HT-29 mean cell diameter from 12 to 15 microns (Fig. S5, *A* and *B*) with a significant increase in cell populations with increased fluorescent intensity from propidium iodide staining (Fig. 6*A*). *MTAP*^*−/−*^ HT-29 cells treated with AG-270 for 5 days accumulated in the G2 phase of the cell cycle (3.7-fold increase, *p* < 0.0001). Similarly, WT HT-29 cells treated with the combination of MTDIA and AG-270 had equivalent increases in G2 (5.8-fold increase, *p* < 0.0001) similar to that of the *MTAP*^*−/−*^ cells treated with AG-270. In WT cells, direct inhibition of PRMT5 with GSK3326595 caused similar G2 cell cycle arrest as combination MTDIA and AG-270 treatment (*p* = 0.2990) but in *MTAP*^*−/−*^ cells the effect was not as pronounced as AG-270 treatment (2.7-fold reduction (*p* = 0.0001, Fig. 6*B*). Cell cycle arrest occurs as a result of downstream effects from PRMT5 inhibition (32, 33).

Treatment of HT-29 cells with the combination of MTDIA and AG-270 increased apoptotic markers, including increases in Annexin V (Fig. 6, *C* and *D*), poly (ADP-ribose) polymerase cleavage, and p53 stabilization (Fig. 6*E*). Caspases 7 and 9 were induced by days 2 and 3 of treatment but not at day 5, and treatment with the pan-caspase inhibitor Q-VD-OPh did not reverse the cell phenotype (Fig. S5, *C* and *D*), suggesting that caspase activation is not the sole mechanism of cell death under these treatment conditions.

PRMT5 inhibition causes many well-characterized changes in RNA splicing (34). The alternative splicing of *MDM4* (human-homolog of Mouse Double-Minute 4) with exon 7 skipping is one consequence of PRMT5 inhibition (30). Cells treated with GSK3326595 or the combination of MTDIA and AG-270 for 0, 3, and 5 days each demonstrated increased alternative splicing beginning at day 3, shown by changes in band intensity between MDM4fl and MDM4s (Fig. 6*F*). Alternative splicing of MDM4 targets the transcript for nonsense-mediated decay, depleting the full-length protein resulting in p53 stabilization and cell apoptosis (29, 30). Both GSK3326595 and the combination of MTDIA and AG-270 treatment increased p53 protein stabilization proportional to the isoform switching of the MDM4 transcript. MDM2, a nonredundant partner of MDM4 responsible for attenuated regulation of p53, is transcriptionally regulated by p53 activation and demonstrated increased expression proportional to p53 expression.

### Xenograft response to MTDIA and AG-270

Toxicity challenge in C57/Bl6 mice revealed that AG-270 (200 mg/kg, once-daily gavage) caused 10% loss of body weight after 15 days. Oral MTDIA alone is reported to be safe and well tolerated in mice (24) and did not cause weight loss alone or enhance weight loss when added at 20 mg/kg in combination with AG-270 (Fig. S6*A*). Liver and kidney function tests including total protein, AST, ALT, BUN, and creatinine levels were within normal levels after 15 days treatment (Fig. S6*B* and Table S4). Histological evaluation of mouse livers, spleens, and gastrointestinal tracts including stomach, small intestine, and large intestine revealed no acute pathology or histological changes consistent with low toxicities (Table S5, histology report, Supplemental data, pp. 12–17). Therefore, MTDIA and AG-270 combination therapy is not acutely toxic to mice.

**Figure 7:**
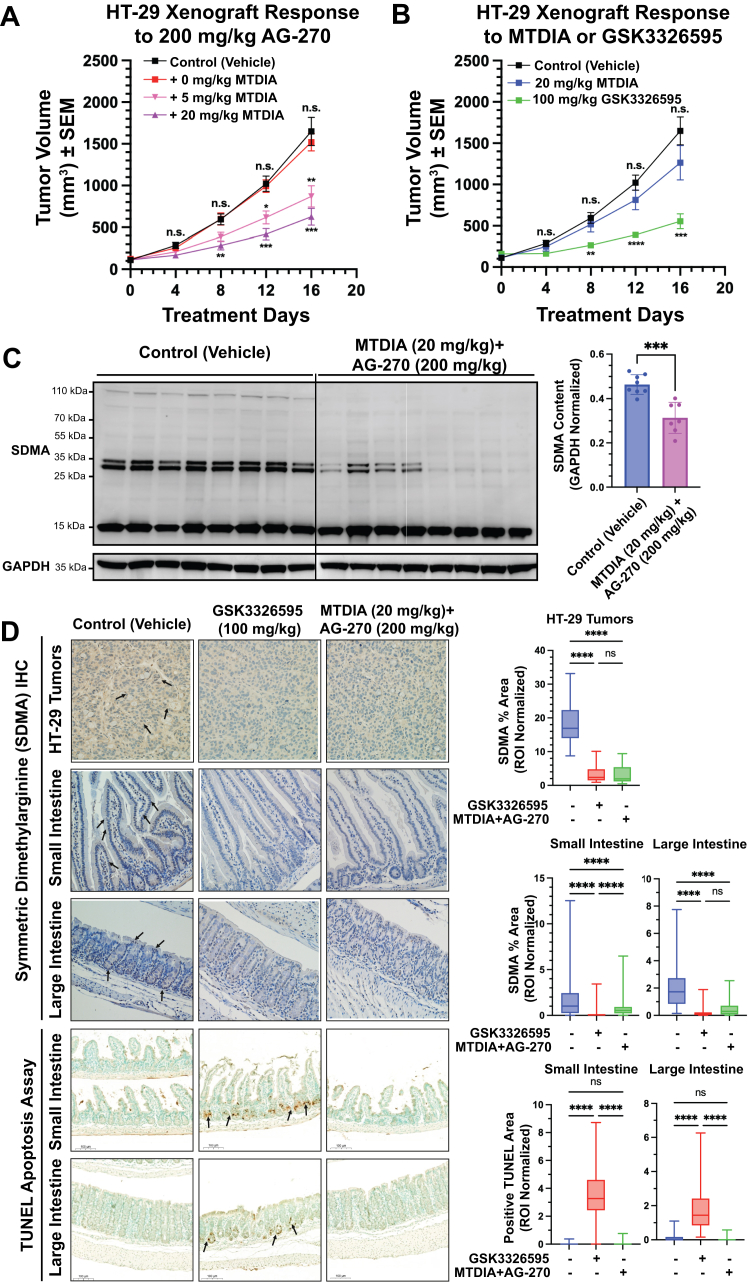
**Xenograft CRC and normal gastrointestinal tissue responses to MTDIA and AG-270 treatment.***A* and *B*, growth curves of WT HT-29 subcutaneous murine xenographs implanted bilaterally in the flanks of NOD/SID mice. Tumors measured *via* digital calipers (n = 12 tumors per condition). *C*, Western blotting of eight control and MTDIA (20 mg/kg) + AG-270 (200 mg/kg) tumors after 20 days treatment. Quantification normalized to GAPDH and performed in ImageJ. *D*, immunohistochemical analysis of SDMA and TUNEL apoptosis content in the HT-29 tumors from NOD/SCID mice and normal small and large intestinal Swiss Roll sections from C57/Bl6 mice after 20 and 15 days of treatment, respectively. Tumor samples were randomly imaged and quantification of SDMA intensity performed by selection of ten regions of interest (ROIs) approximately 150 μm^2^ in area per image with n = 16 imaged per treatment. Small and large intestinal sections were quantified by selecting epithelial cells in villi and crypts and weighting each ROI by total area. Scale bars represent 100 μm length. MTDIA, methylthio-DADMe-immucilin-A; SDMA, symmetric dimethylarginine.

The *in vivo* efficacy of MTDIA and AG-270 combination treatment was evaluated using a murine xenograft model. HT-29 cells were implanted into NOD/SCID mice, and tumors were allowed to grow for 4 days before treatment. AG-270 as a single agent did not reduce tumor growth rate at a dose of 100 or 200 mg/kg. Similarly, MTDIA alone at 20 mg/kg had no significant effect on HT-29 tumor growth. However, addition of MTDIA in the drinking water in mice treated with AG-270 caused a dose-dependent decrease in tumor growth. At day 16, tumor growth was inhibited by 38% and 66% at 5 and 20 mg/kg of MTDIA, respectively (Fig. 7*A*). Treatment with GSK3326595 at 100 mg/kg caused a 76% tumor growth inhibition, similar to MTDIA and AG-270 combination therapy (Fig. 7*B*) (35). Measurement of excised tumors after treatment day 20 confirmed the inhibition during their growth *in vivo* (Fig. S6*D*).

Western blots of tumor protein extracts treated with MTDIA (20 mg/kg) and AG-270 (200 mg/kg) demonstrated a relative decrease of 1.5 ± 0.1-fold SDMA signal intensity (*p* = 0.0002) across tumor samples (Fig. 7*C*). Paraffin-embedded HT-29 tumor tissue samples were evaluated with SDMA-specific antibodies. Tumors treated with MTDIA (20 mg/kg) and AG-270 (200 mg/kg) were decreased 6 ± 1-fold in SDMA content (*p* < 0.0001), equivalent to tumors treated with GSK3326595 (100 mg/kg, 5.0 ± 1.0, *p* < 0.0001). There was no significant difference between GSK3326595 treatment and combination MTDIA and AG-270 treatment (*p* = 0.4390, Fig. 7*D*).

Toxicity analysis for agents also included histopathology of Swiss roll intestinal tissue sections from C57/Bl6 mice treated with MTDIA (20 mg/kg) and AG-270 (200 mg/kg) or with GSK3326595 (100 mg/kg) for 15 days. No histological changes to the villous structures were observed across all samples. Immunohistochemistry revealed significant decreases in SDMA staining intensity across all gut sections in both GSK3326595 and combination MTDIA and AG-270 treated mice (Fig. 7*D*). Sections were stained for markers of apoptosis using the TUNEL assay, revealing significant apoptosis present in gastrointestinal tract crypts of mice treated with GSK3326595, but not those treated with MTDIA and AG-270 (Fig. 7*D*). Apoptosis was localized to the progenitor stem cell compartments in the lower portions of intestinal crypts in the duodenum, jejunum, and ileum and the glandular structures of the colon. No apoptosis was noted in the epithelium further up the length of the villous structures. Therefore, while GSK3326595 induced significant apoptosis in normal intestinal and colonic tissue, the combination of MTDIA and AG-270 did not, although both regimens were equally effective in reducing tumor growth through comparable loss of PRMT5 activity.

## Discussion

Catalytic inhibition of MTAP with MTDIA recapitulates the metabolic phenotype of *MTAP*^*−/−*^ cells and mimics the synthetic lethal phenotype in combination with the MAT2a inhibitor AG-270. As single agents, MTDIA and AG-270 have no effect on *MTAP*^*+/+*^ cell growth, with IC_50_ values >300 μM, but in combination have 10^4^ to 10^5^ greater potency. Combination MTDIA and AG-270 treatment alters metabolite pool sizes of MTA and SAM, establishing a 40-fold increase in the MTA:SAM ratio. Since SAM and MTA compete for the catalytic site on PRMT5, the synergism results in a >90% inhibition of PRMT5 methyltransferase activity. Inhibition of PRMT5 activity resulting from combination MTDIA and AG-270 treatment causes splicing aberrations, including activation of the MDM4-p53 axis, cell cycle arrest in G2 phase, and cell death in MTAP-competent cells. This response mimics those caused by the PRMT5 inhibitor GSK3326595 and also by MAT2a inhibitors in *MTAP*^*−/−*^ cells. In murine models, combination MTDIA and AG-270 did not induce apoptosis of normal colonic cells but did reduce xenograft growth rates indicating its potential as a cancer therapy. Therefore MTDIA has potential to mimic the synthetic lethal phenotype of *MTAP*^*−/−*^ tumors and expand efficacy of MAT2a combination therapy to *MTAP*^*+/+*^ tumors.

### Synthetic lethality and drug synergy with MTAP, MAT2a, and PRMT5 inhibitors

The interaction between MTAP loss and codeletions in MAT2a or PRMT5 is characterized as synthetic lethal in cancers (2). Here, synthetic lethality is induced systemically and is therefore a mimic of genetic *MTAP*^*−/−*^ in the tumor tissue. The biological effects thus parallel inhibition of PRMT5 and downstream cellular processes.

In *MTAP*^*−/−*^ cancer cell lines, MAT2a inhibitor AG-270 has a reported IC_50_ of 260 nM for growth inhibition (28). Here, AG-270 is not effective on *MTAP*^*+/+*^ HT-29 cells but in combination with MTDIA, the IC_50_ for AG-270 decreases 1000-fold to 228 nM, consistent with its efficacy in *MTAP*^*−/−*^ cells. When AG-270 is used to sensitize cells, the IC_50_ for MTDIA is 56 nM, consistent with literature for MTDIA in the presence of excess MTA (25). Drug synergy did not occur between the PRMT5 inhibitor GSK332659 and MTDIA or AG-270 (Fig. S3, *F* and *G*). GSK3326595 is substrate-competitive with the protein substrates of PRMT5 and binds to PRMT5 at a site distinct from MTA and SAM. Synergy plots demonstrate a switch from synergy to antagonism near the observed IC_50_ for GSK3326595 (IC_50_ = 45 nM, Fig. S3*E*), suggesting that above the IC_50_, growth inhibition is dominated by GSK3326595 binding to PRMT5 independent of MTA levels. However, PRMT5 inhibitors that bind cooperatively with the PRMT5–MTA complex, including MRTX1719 or TNG462, would be expected to synergize with MTDIA without the need for MAT2a inhibition in cells (36). The combination of MTDIA with an MTA-cooperative PRMT5 inhibitor is found to be more toxic against the cancers than the host.

### Metabolic regulation of PRMT5 activity

The MTAP-MAT2a-PRMT5 biochemical axis ultimately converges on inhibition of PRMT5. Previous reports demonstrate that MTAP inhibition alone has growth-inhibitory effects in select cell lines in culture (25), xenografts (26) and cancer genetic models (24), modulated through the intracellular accumulation of MTA. Both in culture and in tissues, MTA extracellular diffusion attenuates its concentration gradient (37), and in culture this requires media supplementation with MTA for MTDIA tumor cell growth inhibition. However, cell growth inhibition by the combination of MTDIA and AG-270 was not enhanced by supplementation with 20 μM MTA, demonstrating that cellular metabolism is sufficient to generate the full antiproliferative response. In addition, MTA supplementation did not enhance the kinetics of PRMT5 inhibition (Fig. S4*D*), suggesting that the 40-fold increase in MTA:SAM ratio is sufficient to supress PRMT5 activity in the cell.

The catalytic activity of MTAP in cells maintains a low ratio of MTA:SAM. HT-29 cells in culture have an MTA:SAM ratio of 0.0087 ± 0.0035. Even at this ratio, PRMT5 is 26% inhibited by MTA. When the MTA:SAM ratio reaches 0.36 ± 0.04 from the combination of MTDIA and AG-270 treatment, 93% of PRMT5 is inhibited MTA. Western blots of seven CRC cell lines demonstrated that the expression of MTAP positively correlated with the SDMA content in the cell (r = 0.802, R^2^ = 0.644), but the expression of PRMT5 did not (r = −0.025, R^2^ = 0.001, Fig. 5*C*). Therefore, MTAP expression may be an endogenous regulator that modulates the activity of PRMT5 by regulating catabolism and pool size of MTA. Additionally, we note that treatment of cells with the combination of MTDIA and AG-270 increased MAT2a protein level, but not MTAP or PRMT5 in all cell lines (Fig. 5*D*), suggesting cells respond to depleting SAM levels by inducing MAT2a (28). Harnessing homeostatic metabolism to shift metabolite ratios in favor of MTA pool size alters PRMT5 activity and presents a useful approach to the design of cancer therapeutics.

### MTAP inhibition enhances MAT2a inhibitors in animal models

The reported efficacy of AG-270 in mouse xenograft models required large doses (200 mg/kg) to achieve significant efficacy in *MTAP*^*−/−*^ xenograft studies, but these doses had little effects on *MTAP*^*+/+*^ cells (38). Mouse toxicity, as indicated by weight loss, was observed even at 100 mg/kg AG-270 treatment. MTDIA alone or in addition to AG-270 treatment caused no additional weight loss or liver or renal toxicities (Fig. S6, *A* and *B* and Table S4). Here, *MTAP*^*+/+*^ tumors were sensitized to AG-270 by MTDIA inhibitor treatment to produce an MTAP^−/−^ phenotype. As the majority of human tumors are *MTAP*^*+/+*^, including approximately 98% of colorectal tumors, MTDIA can be an effective strategy for sensitizing them to MAT2a inhibitors (39). The underlying mechanism is by MTDIA causing accumulation of MTA in tissues regardless of tumor MTAP genotype due to the loss of MTA catabolism in all cells (24, 26), thereby enhancing the MTA:SAM ratio, inhibiting PRMT5 activity, and providing dose-dependent sensitivity to MAT2a inhibitors.

### PRMT5 as a therapeutic target for CRC

PRMT5 has been identified as a cancer target for its overexpression in lung, gastric, bladder, blood, breast, lung, and ovarian cancers and is correlated with poorer patient prognoses (40). More recently, PRMT5 is an emerging key target for the treatment of colorectal cancers (16, 17, 41). The combination of MTDIA and AG-270 inhibits the PRMT5-dependent alternative splicing of MDM4 and increases p53 protein, proposed to cause tumor growth arrest and apoptosis (29). PRMT5 has also been implicated in CRC progression through its methylation of SMAD and AKT kinase, regulators of cell division and tumorigenesis in CRC (16, 41).

As the combination MTDIA and AG-270 treatment converges on PRMT5 inhibition, downstream anticancer effects are predicted to mimic those from PRMT5 inhibitors, but the effects are also distinguishable. Importantly, an advantage of combined MTAP and MAT2a inhibition relative to GSK3326595 was found in the histology of normal gut tissue from mice treated by both approaches (Fig. 7*D*). GSK3326595, directly targeting PRMT5, induced apoptosis in normal gut crypt cells but this was not detected with the MTAP + MAT2a inhibitor combination despite inducing comparable loss of PRMT5 activity. The importance is emphasized by reports that in clinical trials with GSK3326595, there were grade 3 or 4 adverse events including gastrointestinal toxicity (42). The underlying cause of this toxicity has not yet been resolved. Additionally in our investigation of tolerability, MTDIA and AG-270 in combination did not exhibit any significant heme-based, liver, or renal toxicities in C57/Bl6 mice after 2 weeks of once-daily drug administration (Table S4), emphasizing further potential advantages of combination MTDIA and AG-270 therapy over PRMT5 inhibitors in human trials. Thus, the lack of detectable alterations in histopathology and in apoptosis in response to MTDIA and AG-270 *in vivo* despite inhibition of tumor growth is clear evidence for limited to no toxicity in the normal tissue, in direct contrast to the reported toxicities of GSK3326595.

### MTAP and MTDIA development potential

In some tumors, homozygous MTAP deletions are associated with poorer survival (43, 44, 45). Additionally, MTAP is reported to have tumor-suppressor functions unrelated to its enzymatic functions when reintroduced to tumors (46). Therefore, our data suggest that the use of the MTAP inhibitor MTDIA in *MTAP*^*+/+*^ tumors would conserve the noncatalytic tumor-suppressive functions while still accumulating MTA that stimulates additional tumor suppressive functions through PRMT5 inhibition. MTDIA is well tolerated, is orally active, and is efficient as an *in vivo* MTAP inhibitor. Mouse studies here predict efficacy as an anticancer agent in combination with MAT2a inhibitors for cancers of *MTAP*^*+/+*^ and *MTAP*^*−/−*^ genotypes. Supporting evidence that MTDIA could enhance anticancer effects even in *MTAP*^*−/−*^ genotypes is found by comparing tumor growth inhibition by single-agent MTDIA treatment in *MTAP*^*+/+*^ and *MTAP*^*−/−*^ lung carcinoma cell lines *in vivo* (26).

Here, we demonstrate the combination of MTDIA and AG-270 treatment has significant potential over GSK3326595 by reducing normal gut epithelial cell death. Further, this combined therapy has significant potential to be effective in colon cancers as well as in cancers at other sites that are sensitive to PRMT5-mediated tumorigenesis. The combination with MAT2a inhibitors or MTA-cooperative PRMT5 inhibitors for further enhancement of anticancer activity will be important in the clinical development of MTDIA.

## Experimental procedures

### Cell culture

All primary cell lines were acquired from the American Type Culture Collection (ATCC): HT-29 (ATCC no. HTB-38), HCT-116 (ATCC no. CCL-247), LoVo (ATCC no. CCL-229), SW-948 (ATCC no. CCL-237), LS-123 (ATCC no. CCL-255), SK-Co-1 (ATCC no. HTB-39), SW-837 (ATCC no. CCL-235), and T84 (ATCC no. CCL-248). Cell lines were cultured in Gibco McCoy5a media (Thermo Fisher Scientific 16600082) supplemented with 10% fetal bovine serum (Thermo Fisher Scientific 16000069) and 1% penicillin/streptomycin (Cytiva SV30010) at 37 °C in 5% CO_2_ atmosphere using 10 cm dishes or 6-well plates treated for tissue culture applications. Two isogenic *MTAP*^*−/−*^ HT-29 cell line clones were purchased from GenScript and their MTAP status was verified by Western blotting (Fig. S1 and Table S3). All cells were passaged using 0.25% trypsin containing EDTA (Thermo Fisher Scientific 25200056) through up to 25 passages. Cells were seeded at 15 to 20% confluency and passaged or harvested for further use at 80 to 90% confluency.

### Drug synthesis and applications

MTDIA was synthesized as previously described (47, 48). MTDIA phosphate salt was dissolved in water and sterilized by ultrafiltration for cell culture. MTDIA concentration was determined by spectrophotometry (MTDIA ϵ = 8.5 cm^−1^ mM^−1^ at 275 nm). MTDIA for mouse experiments was dissolved in sterile drinking water, and the average dose targeted was calibrated to the water consumption of mice as described previously (24). AG-270 was synthesized by published methods (28). AG-270 was administered as a once-daily oral gavage in a vehicle containing 6.7% AFFINISOL hydroxypropylmethyl cellulose HME 15LV hypromellose, 1% PVPk30, 2% D-α-tocopheryl polyethylene glycol succinate, 0.1% simethicone as described (28). GSK3326595 was synthesized according to published methods and administered as a once-daily oral gavage in distilled water (49).

### Growth inhibition assays

A 6-well plate was seeded with 1 × 10^4^ HT-29 cells per well. Growth assays were initiated by exchanging media to contain 1 μM MTDIA, 1 μM AG-270, and/or 20 μM MTA. Cells were trypsinized at 24-h intervals, suspended in fresh media, and an aliquot diluted 1:1 with trypan blue (Thermo Fisher Scientific 15250061). Live cells were visualized under brightfield microscopy and counted in triplicate with a hemacytometer. Cell proliferation curves were generated with GraphPad Prism software version 9.0.0 for Macintosh, GraphPad Software, www.graphpad.com.

### IC_50_ synergy experiments

Each well of a 96-well plate was seeded with 1 × 10^3^ HT-29 cells. Cells were treated with MTDIA and AG-270 at concentrations ranging from 1 nM to 10 μM. After 5 days of treatment, the media was aspirated and replaced with 110 μl of media containing 10% WST-1 reagent (Abcam ab155902). Plates were then incubated at 37 °C for 2 to 3 h for optimal color development of the WST-1 reagent before transferring 100 μl to a fresh 96-well plate for analysis (25 °C at 440 nm). Corrected absorbances were plotted as a function of concentration and calculated IC_50_ values interpolated from four replicates. Growth inhibition curves were generated with GraphPad Prism software, and synergy analyses were performed with SynergyFinder3.0 (50).

### Metabolite extraction and quantification

HT-29 or HCT-116 cells were seeded at 1 × 10^6^ cells per 10 cm dish and grown to 70 to 80% confluency. Metabolic effects of drug treatment were analyzed by exchanging the media to contain 1 μM MTDIA, 1 μM AG-270, or a combination of MTDIA and AG-270 followed by 24 h incubation. Media (500 μl) was stored for further analysis and the remainder was aspirated. The cells were placed on ice and washed with 1 ml cold 1× PBS (4 mM KH_2_PO_4_, 155 mM NaCl, pH 7.4), followed by lysis with 1 ml of ice-cold extraction buffer (40% acetonitrile, 40% methanol, 20% H_2_O, 0.1% formic acid) containing an internal standard of SAM (methyl-*d*_*3*_, Santa Cruz sc-481746). Samples were transferred to Eppendorf tubes, the cellular debris removed by centrifugation and 900 μl of the supernatant dried overnight in a SpeedVac evaporator. Dried samples were resuspended in 40 μl of H_2_O and injected into an Agilent 6490 triple quadrupole mass 148 spectrometer (LC-MS). Detailed methods are provided as Supporting information (Tables S1 and S2).

### Protein extraction and Western blotting

Cells were lysed with 1× radioimmunoprecipitation assay buffer (25 mM Tris–HCl pH 7.6, 150 mM NaCl, 1% NP-40, 1% sodium deoxycholate, 0.15% SDS) containing protease and phosphatase inhibitor (Thermo Fisher Scientific 78429). Sample protein concentration was quantified using a bicinchoninic acid assay (Thermo Fisher Scientific 23227) and normalized to a target concentration of 1 μg protein per μL before SDS preparation. Five to 20 μg of protein was added to SDS containing 5% β-mercaptoethanol, incubated at 100 °C for 5 min, and loaded onto PAGE gels containing 10% Criterion XT Bis-Tris polyacrylamide gel. Following electrophoresis, the fractionated proteins were transferred to a PVDF membranes using iBlot2 semidry transfer (Thermo Fisher Scientific IB24001) at 10 V for 5 min. After transfer, membranes were blocked in 1% wt/vol ECL Prime blocking reagent (Thermo Fisher Scientific 000105) in 0.1% PBS-T for 1 h at room temperature then rinsed with 0.1% PBS-T for 5 min. Primary antibodies for SDMA (CST#13222), GAPDH (ab181602), ADMA (CST#13522), trimethyl lysine (CST#14680), PRMT5 (ab151321), MTAP (ab126770), MAT2a (ab177484), full-length and cleaved poly (ADP-ribose) polymerase (CST#9542 and CST#5625), caspase-3 (CST#14220), caspase-7 (CST#8438), caspase-9 (CST#9508), p53 (CST#2527) MDM4 (ab190364), and MDM2 (CST#86934) were diluted 1:1000 in cold primary antibody solution containing 2.5% bovine serum albumin wt/vol and 0.025% NaN_3_ in 0.1% PBS-T and incubated at 4 °C overnight. Membranes were washed before incubation with horseradish peroxidase–conjugated secondary antibody (ab205718) in 1% wt/vol ECL Prime blocking reagent at 1:10,000 dilution for 1 h at room temperature. Finally, membranes were washed with 1× PBS and visualized with luminol chemiluminescent reagent (Thermo Fisher Scientific J61462-AK) and visualized with ImageQuant (https://cdn.cytivalifesciences.com/api/public/content/digi-14438-original) LAS 4000 mini luminescent image analyzer.

### Cell cycle and apoptosis analysis

Cells were seeded at 10^5^ cells per well in 6-well dishes and treated with MTDIA and/or AG-270 for 5 days. Floating and adherent cells then were collected separately, and apoptosis assayed with Annexin V-FITC ApoTracker Green assay (BioLegend 427402) according to the manufacturer’s protocol. Growth arrest was assessed by fixing cells in cold ethanol for 15 min and staining with ViaStain PI Cell Cycle Kit (CSK-0112) according to manufacturer’s protocol. Cells were analyzed on an LSRII flow cytometer (Becton Dickson) and data were analyzed using FlowJo (https://www.flowjo.com) (TreeStar) and Graphpad Prism.

### Determination of MDM4 alternative splicing

HT-29 cells were treated with GSK3326595 or a combination MTDIA and AG-270 for 0, 3, or 5 days in 6-well plates. RNA was extracted using Qiagen RNeasy Plus Mini Kit (no. 74134) by the manufacturers’ protocol. Ten microliters of the extract was reverse transcribed to cDNA using High-Capacity cDNA Reverse Transcription kit (Applied Biosystems 4374966) and amplified using PCR SuperMix kit (Invitrogen 10572-014) by the manufacturers’ protocol. Primers flanking the MDM4 exon 7 alternative splice site and GAPDH were adapted from Ref (30). Twenty microliters of the PCR product was resolved on a 2% agarose gel and imaged with Bio-Rad ChemiDoc XRS+.

### Murine xenograft implantation and tumor monitoring

Murine studies were conducted under a protocol approved by the Albert Einstein College of Medicine Institutional Animal Care and Use Committee (protocol no. 00001056). Mice homozygous for the NOD.Cg-*Prkdc*^*scid*^ mutation were purchased from The Jackson Laboratory (NOD/SCID, Strain #001303). HT-29 cells were grown in 10 cm dishes to 70 to 80% confluency. Cells were harvested by trypsinization and resuspended in fresh culture media to a concentration of 1 × 10^6^ and mixed 1:1 with 100 μl of Matrigel on ice. The 200 μl cell suspension was injected bilaterally into the shaved subcutaneous flanks of immune-deficient NOD/SCID mice. After 4 days of tumor growth, MTDIA was added to the drinking water to achieve a daily dose of 20 mg/kg of animal body weight. AG-270 was administered once-daily (QD) by oral gavage at 200 mg/kg, and GSK3326595 was administered by oral gavage QD at 100 mg/kg.

Tumors were measured every 4 days by digital caliper measurements and volume calculated (Volume = ((Width)^2^ ⨉ Length)/2) until the tumor volumes approached the Institutional Animal Care and Use Committee–approved limits at approximately 20 days. Animals were sacrificed 4 h postfinal drug dose by CO_2_ asphyxiation, followed by cervical dislocation. Tumors were excised, measured by digital calipers, and portions frozen in liquid nitrogen or formalin-fixed and paraffin-embedding.

### Immunohistochemistry

Tissues were fixed in 10% formalin for 72 h then in 70% ethanol before paraffin-embedding and sectioning by the Albert Einstein Histopathology Core. Sections were dewaxed at 60 °C for 1 h, washed with xylene and ethanol, and endogenous peroxidases quenched with hydrogen peroxide. Rehydrated and quenched slides were blocked with 10% normal goat serum for 1 h at room temperature in a humidified chamber. Slides were then incubated with rabbit anti-SDMA antibody (MBS619480) at 1:400 dilution overnight at 4 °C in the same chamber. Slides were rinsed in 1× PBS three times for 5 min before incubation for 1 h at room temperature with biotinylated goat anti-rabbit secondary antibody (1:100). Sections were then rinsed in 1× PBS three times for 5 min and then were incubated with ABC solution (Vector lab, Elite ABC) for 30 min. Signal was developed with 3,3-diaminobenzidine substrate (10–50 sec incubation) until sufficient brown color developed. Slides were then washed in 1× PBS, counterstained with Harris Hematoxylin, and dehydrated and mounted for analysis.

### Statistical analyses

Statistical analysis utilized with GraphPad Prism software v.9.0. Data are presented as mean ± SD unless otherwise stated. The significance of cell line experiments was determined by Student *t* test (two-tailed, equal variance) or one-way ANOVA. Tumor growth curves were analyzed using two-way ANOVA.

## Conclusion

The synthetic lethal relationship between *MTAP* homozygous deletions and codeletions in *MAT2a* have stimulated the development of MAT2a inhibitors for treatment of the 15% of human tumors that are *MTAP*^*−/−*^. The development of MTDIA as a tight-binding transition state analog of MTAP renders cells MTAP-deficient, which phenocopies the metabolic signature of genetically *MTAP*^*−/−*^ tumors and sensitizes *MTAP*^*+/+*^ cells to MAT2a inhibition *via* specific inhibition of PRMT5. As the *MTAP*^*−/−*^ genotype is rare in CRCs, this approach establishes a new therapeutic window for CRCs treatment with MAT2a inhibitors, in addition to treatment of tumors at other sites with *MTAP*^*+/+*^ genotypes. The combination of MTDIA and AG-270 treatment raises the intracellular MTA:SAM ratio to exert metabolic regulation of PRMT5 activity. Inhibition of PRMT5 causes alternative splicing events, activation of cell cycle arrest, and apoptosis leading to growth inhibition in both *in vitro* cell culture and *in vivo* human xenograft models. Importantly, no significant toxicities or cell death was observed in the normal guts mucosa of mice treated with MTDIA and AG-270 in contrast to those treated with PRMT5 inhibitor GSK3326595, highlighting an additional benefit of this treatment and the essential function of PRMT5 in cancers. Further investigation into the genetic and biochemical mechanisms and therapeutic strategies are warranted.

## Data availability

Data supporting this work are disclosed within the manuscript and/or are available in the supplementary materials available online. G. T. B. *et al*. “Combined inhibition of MTAP and MAT2a mimics synthetic lethality in tumor models *via* PRMT5 inhibition” *JBC* submission, September 2023.

## Supporting information

This article contains supporting information.

## Conflict of interest

The authors declare that they have no conflicts of interest with the contents of this article.
