# Supplementary material for: Combined inhibition of MTAP and MAT2a mimics synthetic lethality in tumor models via PRMT5 inhibition
Source: J Biol Chem. 2023 Nov 23;300(1):105492. doi: 10.1016/j.jbc.2023.105492 (PMC10770533; doi:10.1016/j.jbc.2023.105492)
Supplement: Supporting Tables S1–S5 and Figures S1–S6 [file mmc1.docx]

# Combined inhibition of MTAP and MAT2a mimics synthetic lethality in tumor models via PRMT5 inhibition

Gabriel T. Bedard^1^, Nord Gilaj^1,2^, Karina Peregrina^3^, Isabella Brew^1^, Elena Tosti^4^, Karl Shaffer^5^, Peter C. Tyler^5^, Winfried Edelmann^4^, Leonard H. Augenlicht^3^, and Vern L. Schramm^1^

^1^Department of Biochemistry, Albert Einstein College of Medicine, Bronx NY 10461;

^2^Lehman College, Bronx NY 10468;

^3^Department of Oncology, Department of Medicine, Department of Cell Biology, Albert Einstein College of Medicine, Bronx NY 10461;

^4^Department of Cell Biology, Department of Genetics, Albert Einstein College of Medicine, Bronx NY 10461.

^5^Ferrier Research Institute, Victoria University of Wellington, Lower Hutt, 5010, New Zealand,

**Supplemental Information**

**Index:**

**Supplemental Methods**

Mass spectrometry …………………………………………………………………………. Page 2

Table S1: Chromatographic method for tandem mass spectrometry ………………………. Page 2

Table S2: Mass identities for methionine-related metabolites and drug compounds ……… Page 2

**Supplemental Figures, Tables, and Reports**

Supplemental Figure 1 ……………………………………………………………………. Page 3

Table S3: gRNA sequences for CRISPR-mediated MTAP knockout ……………………. Page 3

Supplemental Figure 2 ……………………………………………………………………. Page 4

Supplemental Figure 3 ……………………………………………………………………. Page 5

Supplemental Figure 4 ……………………………………………………………………. Page 7

Supplemental Figure 5 ……………………………………………………………………. Page 8

Supplemental Figure 6 ……………………………………………………………………. Page 9

Table S4: Blood chemistries and counts in C57/Bl6 mice ………………………………... Page 10

Table S5: Histopathology Animal Treatment Assignments ……………………………… Page 11

Histopathology Report ……………………………………………...………………….…. Page 12

**Supplemental Methods:**

Mass spectrometric determination of metabolite quantities: High-performance liquid chromatography (HPLC) with tandem detection by mass spectrometry (LC-MS) was conducted on an Agilent Technologies 1200 system coupled with 6410 triple quadrupole mass spectrometer. Dried cell and tissue extracts were resuspended in 40 μL of 1:1 mixture of 0.1% formic acid and acetonitrile and insoluble particles clarified by centrifugation. 10 μL of the supernatant resolved on a Zorbax Rapid Resolution SB-C18 column (2.4 mm x 35 mm, 3.5 μm particle size) pre-equilibrated in 99% buffer A (5 mM Perfluoroheptanoic acid, 6 mM ammonium formate pH 3) with 1% buffer B (100% Acetonitrile). The chromatographic method in Table S1 provided high-throughput separation of samples.

**Table S1: Chromatographic method for tandem mass spectrometry**

| **Time Segment (minutes)** | **Flow Rate (mL min^-1^)** | **Solvent A (%)** | **Solvent B (%)** |
| --- | --- | --- | --- |
| 0.00 | 0.400 | 99.0 | 1.00 |
| 0.5 | 0.400 | 95.00 | 5.00 |
| 2.50 | 0.400 | 45.00 | 55.00 |
| 3.50 | 0.400 | 0.00 | 100.00 |
| 5.00 | 0.400 | 0.00 | 100.00 |
| 6.50 | 0.400 | 99.00 | 1.00 |
| 7.50 | 0.400 | 99.00 | 1.00 |

The quadrupole was set to positive mode and the first 0.5 minutes of elution sent to waste. Multi-reaction monitoring (MRM) was collected from 0.5 minutes to 7.0 minutes for the masses listed in Table S2. All peaks were validated against standard compounds purchased through Sigma Aldrich.

**Table S2: Mass identities for methionine-related metabolites and drug compounds**

| **Compound** | **Retention Time (min)** | **Collision energy (meV)** | **Parent Ion (m/z)** | **Product Ion 1 (quantifier)** | **Product ion 2 (qualifier)** |
| --- | --- | --- | --- | --- | --- |
| *S*-adenosyl-L-methionine, methyl-*d_3_* | 3.55 | 15 | 402.1 | 301.1 | 249.8 |
| *S*-adenosyl-L-methionine | 3.55 | 15 | 399.1 | 298.1 | 249.8 |
| 5’-methylthioadenosine | 3.14 | 10 | 298.1 | 145.1 | 136.0 |
| *S*-adenosyl-L-homocysteine | 3.47 | 10 | 384.8 | 249.8 | 135.9 |
| Spermine | 3.52 | 10 | 203.3 | 129.9 | 112.9 |
| Methionine | 2.15 | 20 | 150.2 | 74.0 | 61.0 |
| Spermidine | 3.51 | 10 | 146.2 | 112.3 | 111.9 |
| Homocysteine | 2.42 | 15 | 136.1 | 73.2 | 56.1 |
| Putrescine | 3.35 | 5 | 89.1 | 72.1 | 72.1 |
| AG-270 | 4.54 | 40 | 490.1 | 223.1 | 121.1 |
| GSK3326595 | 4.38 | 20 | 453.1 | 189.1 | 146.2 |
| MTDIA | 3.58 | 20 | 294.1 | 148.1 | 82.0 |

**
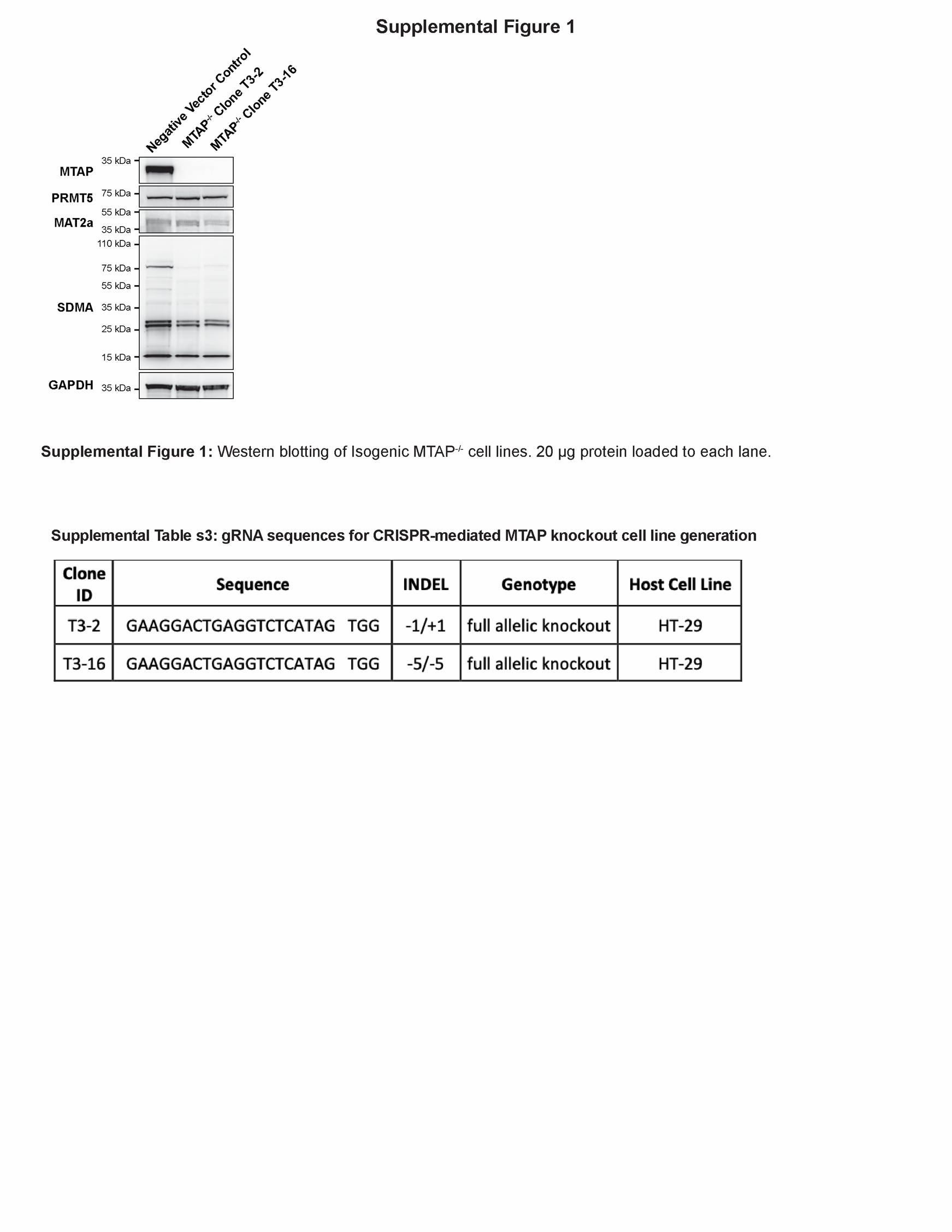
**

**
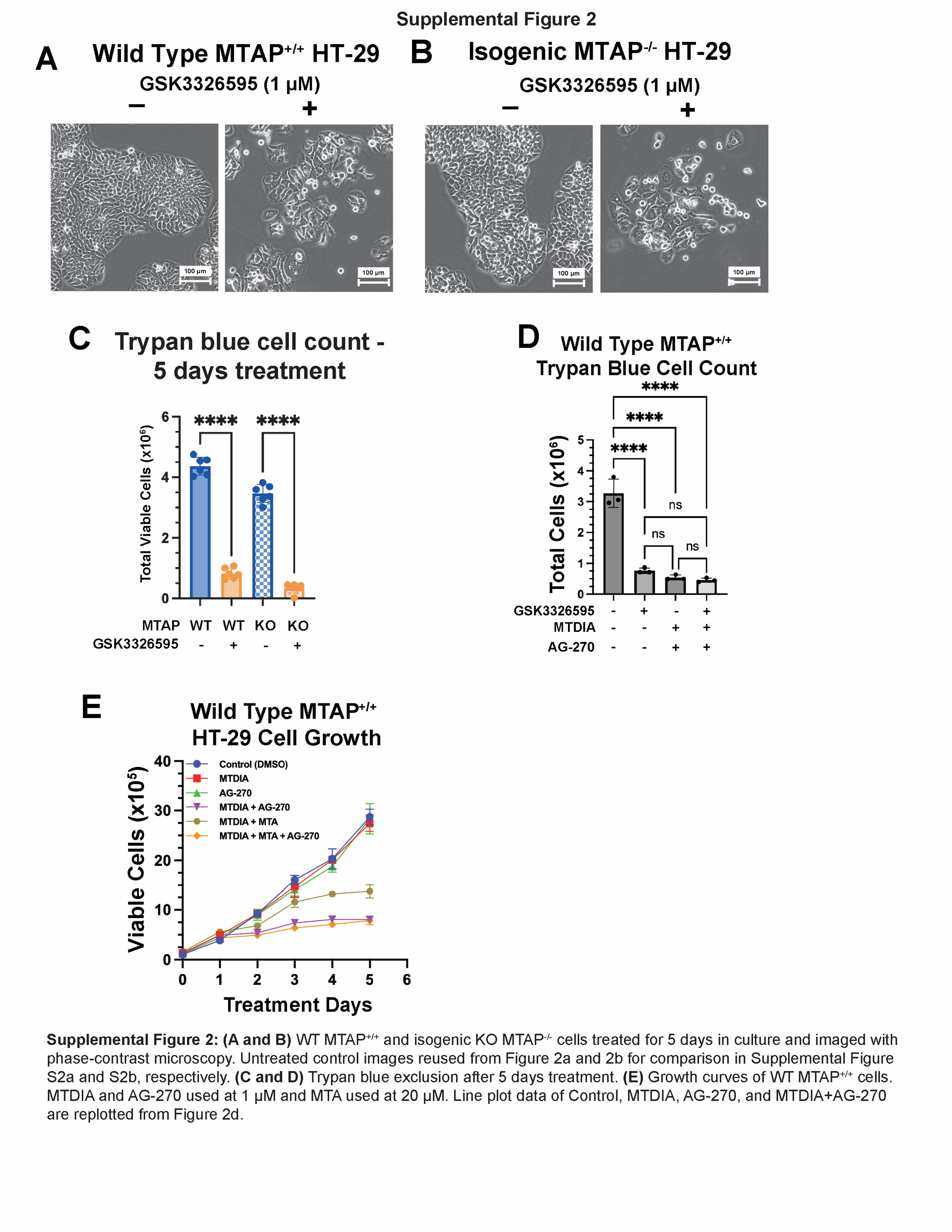
**

**
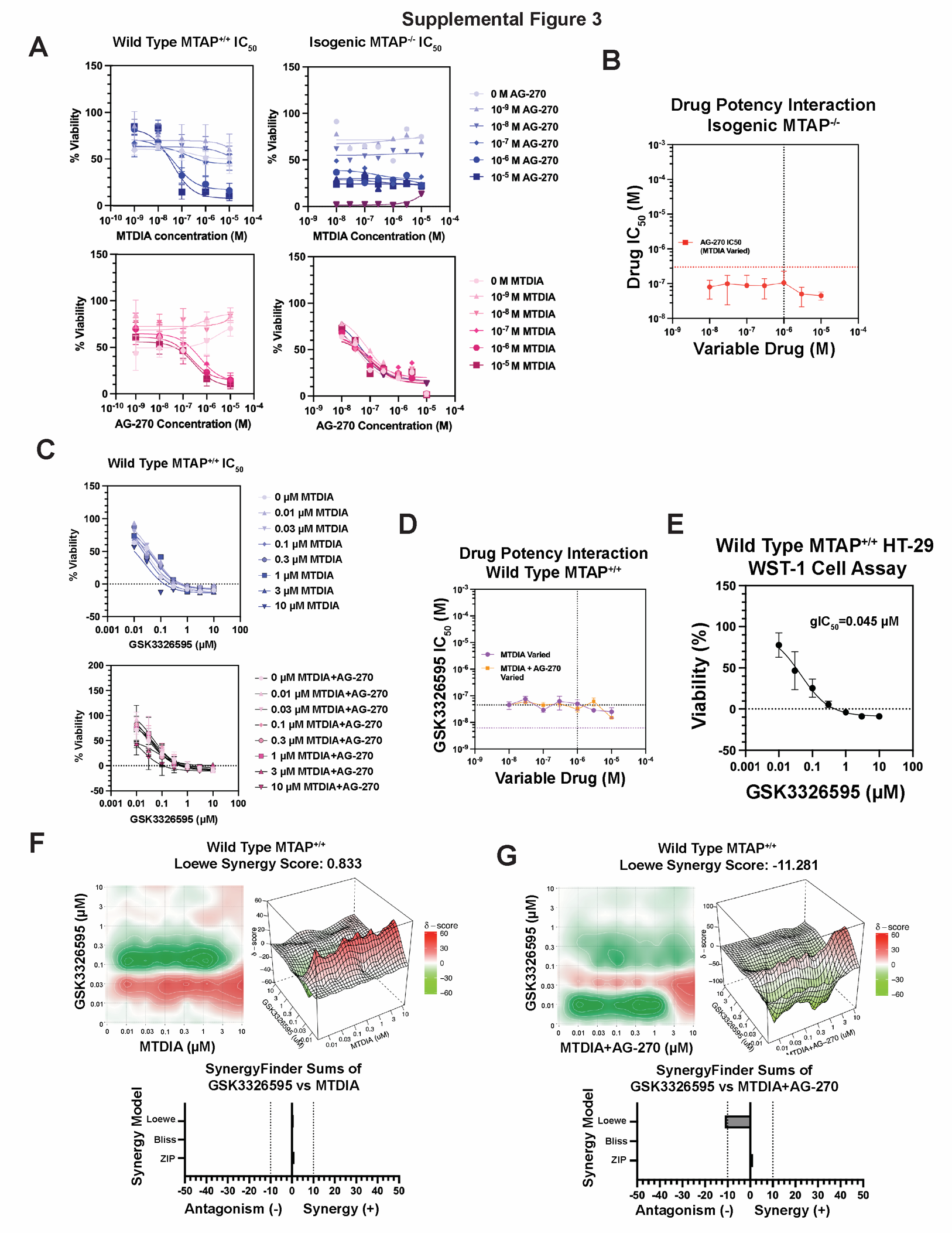
**

**
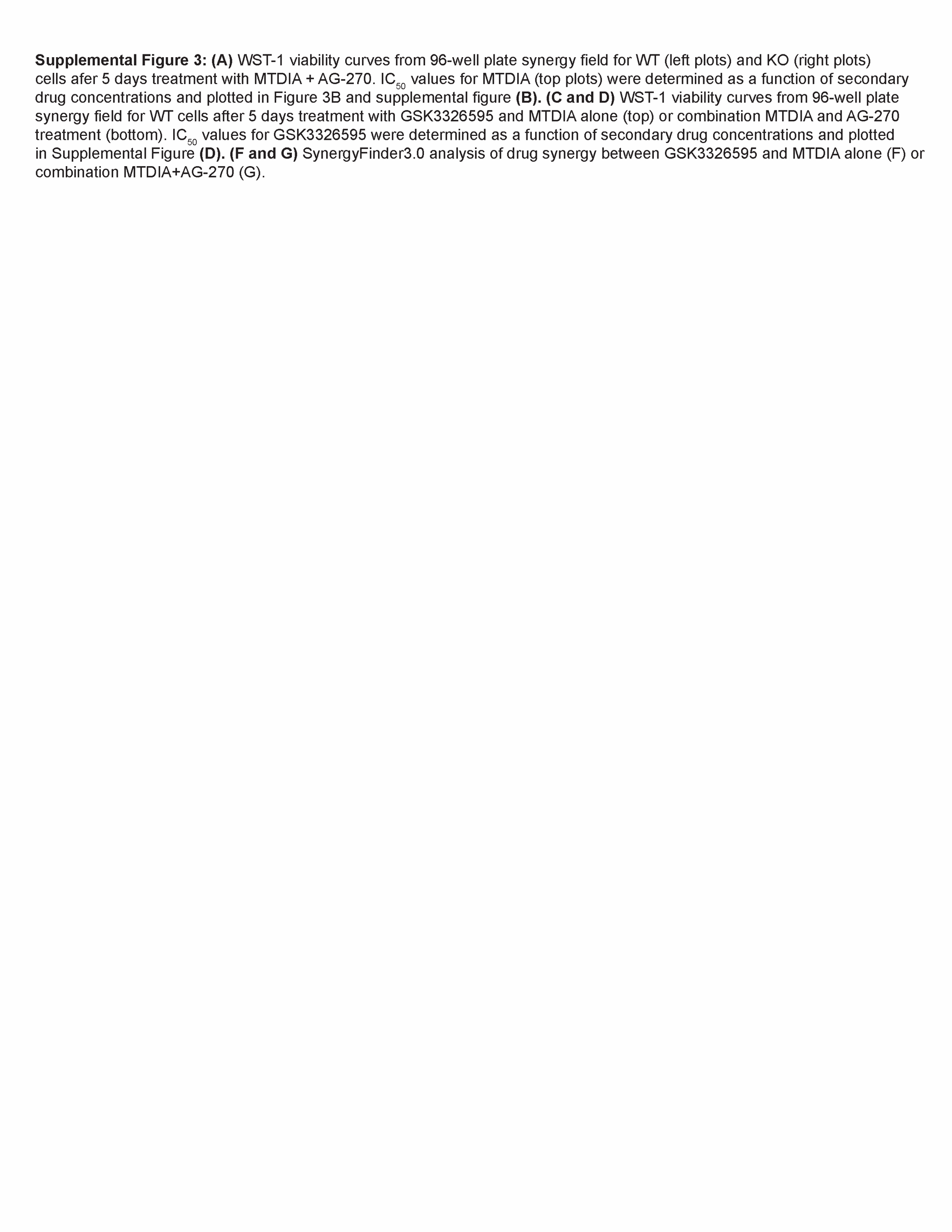
**

**
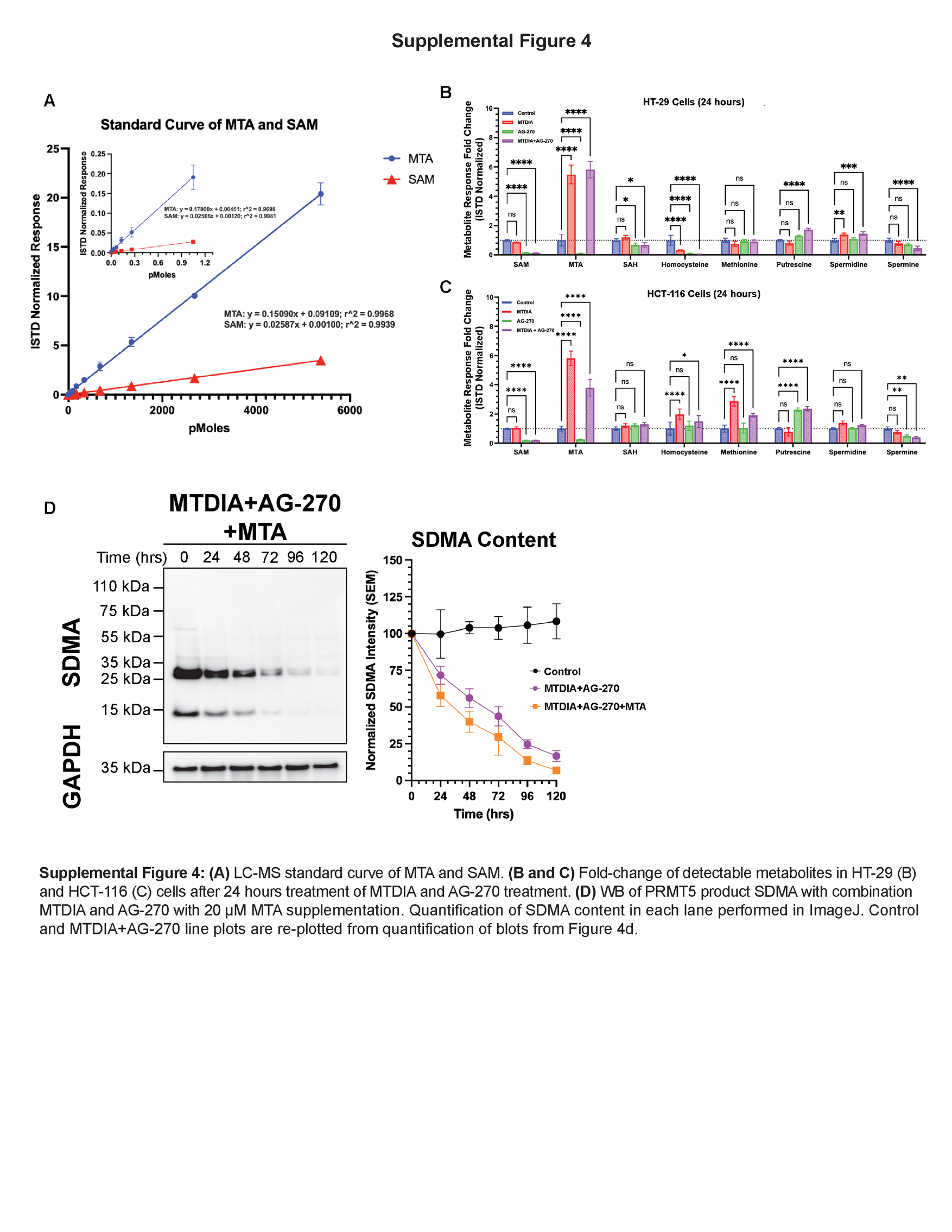
**

**
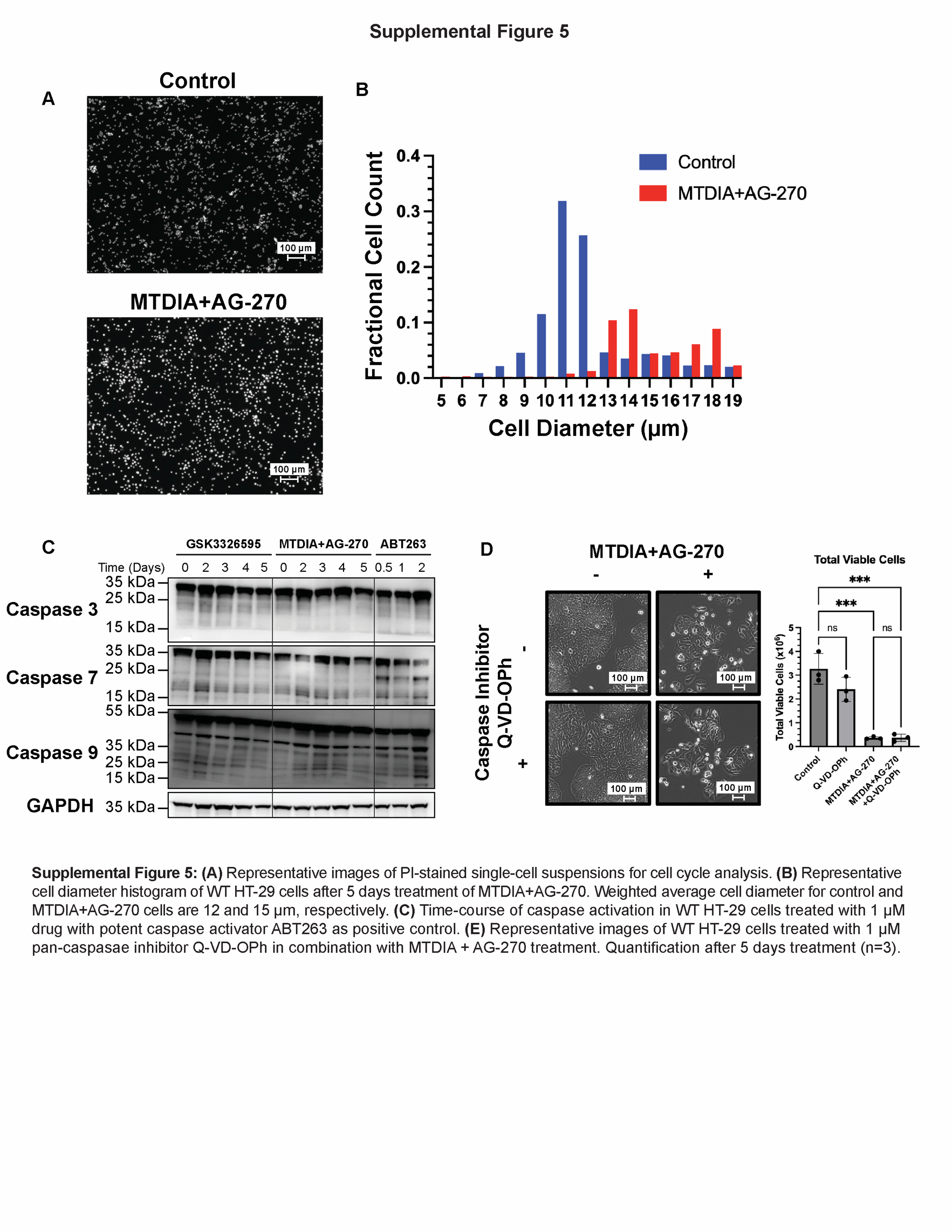
**

**
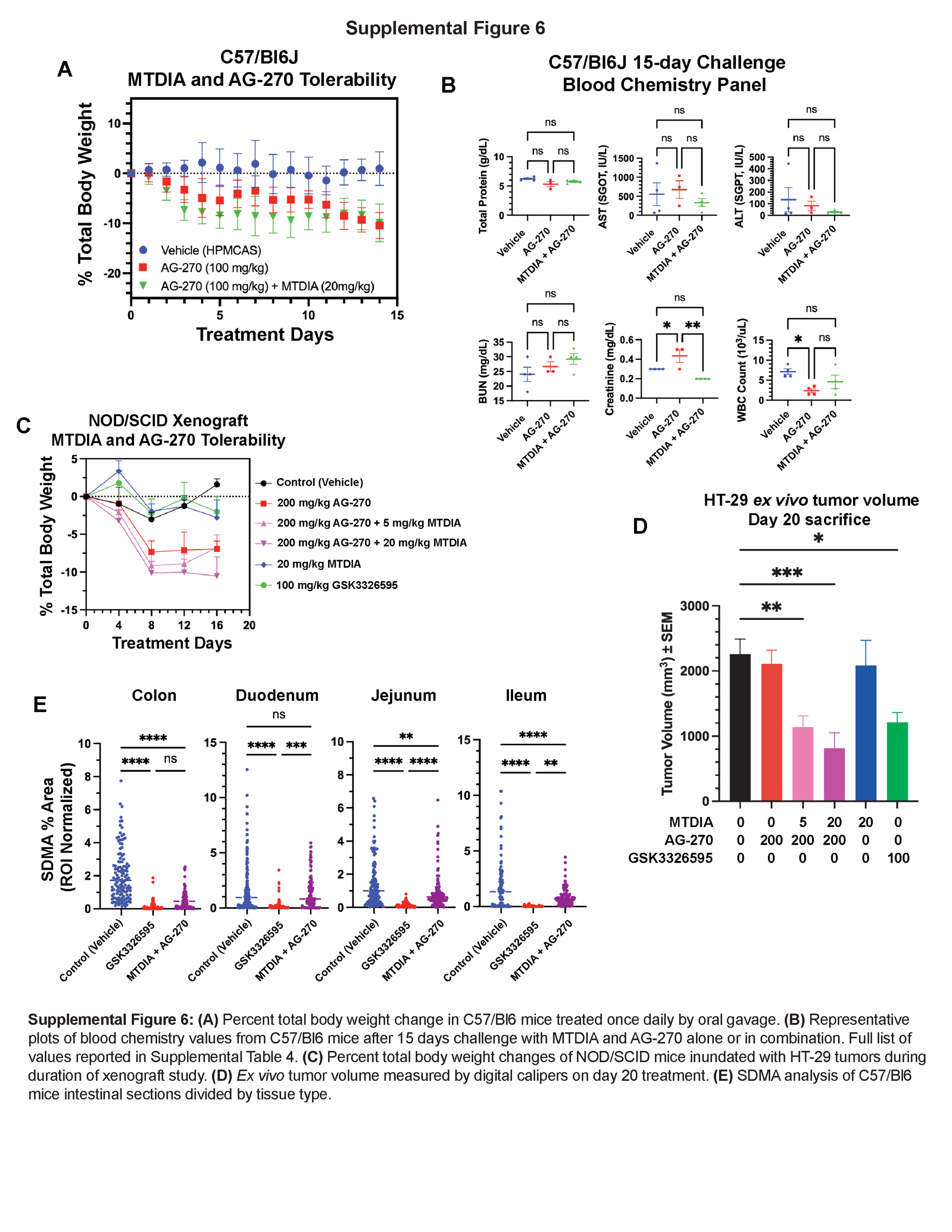
**

**
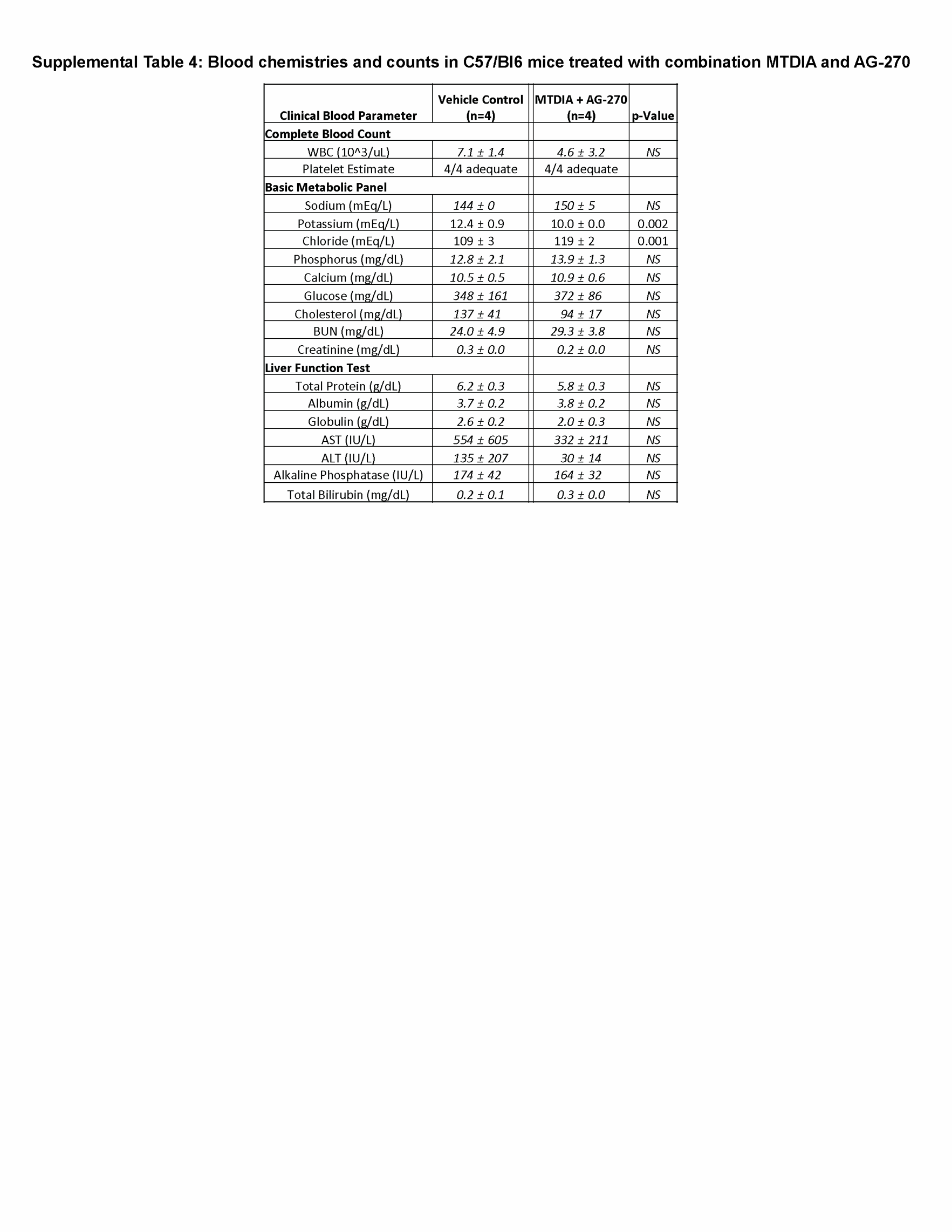
**

**Table S5: Histology Report Mouse Assignment Key**

| **Mouse Number** | **GENDER** | **MTDIA Tx** | **AG-270 Tx** | **GSK-3326595** |
| --- | --- | --- | --- | --- |
| 1 | Male | 0 | 0 - Vehicle | 0 |
| 2 | Male | 0 | 0 - Vehicle | 0 |
| 3 | Female | 0 | 0 - Vehicle | 0 |
| 4 | Female | 0 | 0 - Vehicle | 0 |
| 5 | Male | 0 | 100 | 0 |
| 6 | Male | 0 | 100 | 0 |
| 7 | Female | 0 | 100 | 0 |
| 8 | Female | 0 | 100 | 0 |
| 9 | Male | 20 | 50 | 0 |
| 10 | Male | 20 | 50 | 0 |
| 11 | Male | 20 | 50 | 0 |
| 12 | Female | 20 | 50 | 0 |
| 13 | Female | 20 | 50 | 0 |
| 14 | Female | 20 | 50 | 0 |
| 15 | Male | 20 | 100 | 0 |
| 16 | Male | 20 | 100 | 0 |
| 17 | Male | 20 | 100 | 0 |
| 18 | Female | 20 | 100 | 0 |
| 19 | Female | 20 | 100 | 0 |
| 20 | Female | 20 | 100 | 0 |
| 21 | Male | 20 | 100 | 0 |
| 22 | Male | 20 | 100 | 0 |
| 23 | Male | 20 | 100 | 0 |
| 24 | Female | 20 | 100 | 0 |
| 25 | Female | 20 | 100 | 0 |
| 26 | Female | 20 | 100 | 0 |
| 27 | Male | 0 | 0 | 100 |
| 28 | Female | 0 | 0 | 100 |

# PATHOLOGY REPORT

# HISTOPATHOLOGY AND COMPARATIVE PATHOLOGY FACILITY

## **ALBERT EINSTEIN COLLEGE OF MEDICINE**

Scientific Director/Comparative Pathologist: Dr. Amanda Beck, Amanda.beck@einsteinmed.org

Laboratory Manager: Laura Nanette Ramkissoon, laura.ramkissoon@einsteinmed.org

**Lab: (718) 678-1043** **Pathologist: (718) 678-1111**

**Accession #: HP21-9376**

**Date: 10-11-21**

**Investigator: Karina Peregrina/Edward Chu**

**History:**

27 mice were put on drug tolerance tests with MTDIA-( inhibits growth of human lung, breast, prostate, colon and head and neck cancers) and AG-270 (MAT-2A inhibitor) in combination at 20/50 or 20mg/100mg. On AG-270 alone or GSK alone another drug similar to AG270. M1-4 are control animals only on vehicle.

We would like you to evaluate all the swiss rolls for any anomalies/obvious pathology. We are seeing elevation in liver ez's, and decrease in white blood cells from CBC why we are asking to have a look at all the livers and spleens.

Examine 1-12, 14-28, all livers, all spleens

**Histology:**

The following organs are examined histologically: GI rolls, liver, spleen.

- ***Frequently, GI rolls (particularly small intestine sections) exhibit autolysis and/or collection/tissue preparation artifact that obscures mucosal architecture and precludes complete histologic evaluation.

GI Rolls (Slides 1-12, 14-28)

Slide 1 (M1): The sections examined are within normal histologic limits.

Slide 2 (M2): The sections examined are within normal histologic limits.

Slide 3 (M3): The sections examined are within normal histologic limits.

Slide 4 (M4): The sections examined are within normal histologic limits.

Slide 5 (M5): There is mild to moderate lymphoid hyperplasia within the Peyer’s patches of the small intestine.

Slide 6 (M6): The sections examined are within normal histologic limits.

Slide 7 (M7): The sections examined are within normal histologic limits.

Slide 8 (M8): There is mild lymphoid hyperplasia within the Peyer’s patches of the small intestine.

Slide 9 (M9): The sections examined are within normal histologic limits.

Slide 10 (M10): There is mild lymphoid hyperplasia within the Peyer’s patches of the small intestine.

Slide 11 (M11): There is mild lymphoid hyperplasia within the Peyer’s patches of the small intestine.

Slide 12 (M12): The sections examined are within normal histologic limits.

Slide 14 (M14): The sections examined are within normal histologic limits.

Slide 15 (M15): The sections examined are within normal histologic limits.

Slide 16 (M16): The sections examined are within normal histologic limits.

Slide 17 (M17): The sections examined are within normal histologic limits.

Slide 18 (M18): The sections examined are within normal histologic limits.

Slide 19 (M19): The sections examined are within normal histologic limits.

Slide 20 (M20): The sections examined are within normal histologic limits.

Slide 21 (M21): The sections examined are within normal histologic limits.

Slide 22 (M22): The sections examined are within normal histologic limits.

Slide 23 (M23): There is minimal to mild lymphoid hyperplasia within the Peyer’s patches of the small intestine.

Slide 24 (M24): The sections examined are within normal histologic limits.

Slide 25 (M25): The sections examined are within normal histologic limits.

Slide 26 (M26): The sections examined are within normal histologic limits.

Slide 27 (M27): The sections examined are within normal histologic limits.

Slide 28 (M28): The sections examined are within normal histologic limits.

Livers

Slide 29 (M1): There are occasional small perivascular to random foci of mixed leukocytes.

Slide 35 (M2): There are occasional small random foci of mixed leukocytes admixed with single cell hepatocyte degeneration/necrosis.

Slide 41 (M3): There are occasional small random foci of mixed leukocytes admixed with single cell hepatocyte degeneration/necrosis.

Slide 47 (M4): There are occasional small random foci of mixed leukocytes admixed with single cell hepatocyte degeneration/necrosis.

Slide 53 (M5): There are occasional small random foci of mixed leukocytes admixed with single cell hepatocyte degeneration/necrosis.

Slide 59 (M6): There are occasional small random foci of mixed leukocytes admixed with single cell hepatocyte degeneration/necrosis.

Slide 65 (M7): There are occasional small random foci of mixed leukocytes admixed with single cell hepatocyte degeneration/necrosis.

Slide 71 (M8): There are occasional small random foci of mixed leukocytes admixed with single cell hepatocyte degeneration/necrosis.

Slide 77 (M9): There are occasional small random foci of mixed leukocytes admixed with single cell hepatocyte degeneration/necrosis.

Slide 83 (M10): There are occasional small random foci of mixed leukocytes admixed with single cell hepatocyte degeneration/necrosis.

Slide 89 (M11): There are occasional small random foci of mixed leukocytes admixed with single cell hepatocyte degeneration/necrosis. Multifocally, within one region, there are clusters of hepatocytes exhibiting cytoplasmic vacuolation consistent with lipid.

Slide 95 (M12): There are occasional small random foci of mixed leukocytes admixed with single cell hepatocyte degeneration/necrosis and multifocally, there are clusters of hepatocytes exhibiting cytoplasmic vacuolation consistent with lipid.

Slide 101 (M14): There are occasional small random foci of mixed leukocytes and multifocally, there are clusters of hepatocytes exhibiting cytoplasmic vacuolation consistent with lipid.

Slide 107 (M15): There are occasional small random foci of mixed leukocytes admixed with single cell hepatocyte degeneration/necrosis.

Slide 113 (M16): There are occasional small random foci of mixed leukocytes admixed with single cell hepatocyte degeneration/necrosis.

Slide 119 (M17): There are occasional small random foci of mixed leukocytes admixed with single cell hepatocyte degeneration/necrosis.

Slide 125 (M18): There are occasional small random foci of mixed leukocytes admixed with single cell hepatocyte degeneration/necrosis.

Slide 131 (M19): There are occasional small random foci of mixed leukocytes admixed with single cell hepatocyte degeneration/necrosis.

Slide 137 (M20): There are occasional small random foci of mixed leukocytes admixed with single cell hepatocyte degeneration/necrosis and multifocally, there are clusters of hepatocytes exhibiting cytoplasmic vacuolation consistent with lipid.

Slide 145 (M21): There are occasional small random foci of mixed leukocytes admixed with single cell hepatocyte degeneration/necrosis.

Slide 151 (M22): There are occasional small random foci of mixed leukocytes admixed with single cell hepatocyte degeneration/necrosis.

Slide 157 (M23): There are occasional small random foci of mixed leukocytes admixed with single cell hepatocyte degeneration/necrosis.

Slide 163 (M24): There are occasional small random foci of mixed leukocytes admixed with single cell hepatocyte degeneration/necrosis and multifocally, there are clusters of hepatocytes exhibiting cytoplasmic vacuolation consistent with lipid. There is also multifocal mild oval cell hyperplasia.

Slide 169 (M25): There are occasional small random foci of mixed leukocytes admixed with single cell hepatocyte degeneration/necrosis.

Slide 175 (M26): There are occasional small random foci of mixed leukocytes admixed with single cell hepatocyte degeneration/necrosis.

Slide 181 (M27): There are rare small random foci of mixed leukocytes.

Slide 187 (M28): There are occasional small random foci of mixed leukocytes admixed with single cell hepatocyte degeneration/necrosis.

Spleens

Slide 31 (M1): Within the white pulp, there is minimal to mild lymphoid hyperplasia (expansion of lymphoid follicles with occasional coalescence and formation of germinal centers).

Slide 37 (M2): The section examined is within normal histologic limits.

Slide 43 (M3): The section examined is within normal histologic limits.

Slide 49 (M4): The section examined is within normal histologic limits.

Slide 55 (M5): The section examined is within normal histologic limits.

Slide 61 (M6): The section examined is within normal histologic limits.

Slide 67 (M7): The section examined is within normal histologic limits.

Slide 73 (M8): The section examined is within normal histologic limits.

Slide 79 (M9): Within the white pulp, there is minimal multifocal lymphoid depletion (decreased cellular density within lymphoid follicles).

Slide 85 (M10): Within the white pulp, multifocal lymphoid follicles exhibit hyperplasia, and other exhibit mild lymphoid depletion. Within the red pulp, there is mild intracellular and extracellular dark brown globular pigment.

Slide 91 (M11): Within the white pulp, there is mild multifocal lymphoid depletion.

Slide 97 (M12): The section examined is within normal histologic limits.

Slide 103 (M14): The section examined is within normal histologic limits.

Slide 109 (M15): The section examined is within normal histologic limits.

Slide 115 (M16): Within the red pulp, there is mild intracellular and extracellular dark brown globular pigment.

Slide 121 (M17): The section examined is within normal histologic limits.

Slide 127 (M18): The section examined is within normal histologic limits.

Slide 133 (M19): Within the red pulp, there is mild intracellular and extracellular dark brown globular pigment.

Slide 139 (M20): Within the white pulp, multifocal lymphoid follicles exhibit hyperplasia, and other exhibit mild lymphoid depletion. Within the red pulp, there is mild intracellular and extracellular dark brown globular pigment.

Slide 147 (M21): Within the white pulp, there is minimal multifocal lymphoid depletion.

Slide 153 (M22): Within the white pulp, multifocal lymphoid follicles exhibit hyperplasia, and other exhibit mild lymphoid depletion.

Slide 159 (M23): Within the white pulp, there is minimal to mild multifocal lymphoid depletion, with apoptotic lymphocytes.

Slide 165 (M24): Within the white pulp, there is minimal to mild multifocal lymphoid depletion, with apoptotic lymphocytes.

Slide 171 (M25): The section examined is within normal histologic limits.

Slide 177 (M26): Within the white pulp, there is minimal multifocal lymphoid depletion.

Slide 183 (M27): The section examined is within normal histologic limits.

Slide 189 (M28): Within the red pulp, there is mild intracellular and extracellular golden brown globular pigment.

**Comments:**

GI Rolls: Five samples (M5, M8, M10, M11, M23) had mild (to focally moderate) lymphoid hyperplasia within the Peyer’s patches of the small intestine. Lymphoid hyperplasia can be evident to varying degrees in normal rodents, depending on age, animal health status, and even the plane of section, and is generally considered to be a non-specific reactive or immune response. For the remaining 22 samples (M1-M4, M6, M7, M9, M12-M22, M24-M28), the sections examined were within normal histologic limits. Of note, within the samples submitted, frequent rolls (particularly the small intestine sections) exhibited autolysis and/or collection/tissue preparation artifacts that obscured mucosal architecture and precluded complete histologic evaluation.

Liver: All samples exhibited occasional small random foci of mixed leukocytes, sometimes associated with single cell hepatocyte degeneration/necrosis. This is a common background finding in adult mice. Five samples (M11, M12, M14, M20, M24) had clusters of hepatocytes exhibiting lipidosis, and one sample (M24) had multifocal mild oval cell hyperplasia. Lipidosis (fatty change) may be a spontaneous change or can be associated with metabolic disturbances or toxicity.

Spleen: Histologic findings in the spleens were minimal to mild and non-specific. They included lymphoid hyperplasia (M1), lymphoid depletion with or without apoptotic lymphocytes (M9, M11, M21, M23, M24, M26), or a combination of both lymphoid hyperplasia and depletion (M10, M20, M22). Lymphoid hyperplasia can be evident to varying degrees in normal rodents, depending on age, animal health status, and even the plane of section, and is generally considered to be a non-specific reactive or immune response. Lymphoid depletion can be observed as a spontaneous change in older mice or can occur as a direct treatment-related effect or an indirect effect secondary to weight loss. Five samples (M10, M16, M19, M20, M28) exhibited a small amount of pigment within the splenic red pulp, a common background finding in rodents. The pigment can be hemosiderin, ceroid/lipofuscin and/or melanin, and these may be differentiated with the use of special histochemical stains if desired. For the 15 remaining samples (M2-M8, M12-M15, M17, M18, M25, M27), the sections examined were within normal histologic limits.

****IF ANY OF THIS MATERIAL WILL BE USED IN PUBLICATION, PLEASE INCLUDE THE FOLLOWING STATEMENT IN THE ACKNOWLEDGMENTS:** “Research reported in this publication was supported by the Albert Einstein Cancer Center Support Grant of the National Institutes of Health under award number P30CA013330.”
